# A first-draft human protein-interaction map

**DOI:** 10.1186/gb-2004-5-9-r63

**Published:** 2004-08-13

**Authors:** Ben Lehner, Andrew G Fraser

**Affiliations:** 1 The Wellcome Trust Sanger Institute, Hinxton, Cambridge CB10 1SA, UK

## Abstract

**Background:**

Protein-interaction maps are powerful tools for suggesting the cellular functions of genes. Although large-scale protein-interaction maps have been generated for several invertebrate species, projects of a similar scale have not yet been described for any mammal. Because many physical interactions are conserved between species, it should be possible to infer information about human protein interactions (and hence protein function) using model organism protein-interaction datasets.

**Results:**

Here we describe a network of over 70,000 predicted physical interactions between around 6,200 human proteins generated using the data from lower eukaryotic protein-interaction maps. The physiological relevance of this network is supported by its ability to preferentially connect human proteins that share the same functional annotations, and we show how the network can be used to successfully predict the functions of human proteins. We find that combining interaction datasets from a single organism (but generated using independent assays) and combining interaction datasets from two organisms (but generated using the same assay) are both very effective ways of further improving the accuracy of protein-interaction maps.

**Conclusions:**

The complete network predicts interactions for a third of human genes, including 448 human disease genes and 1,482 genes of unknown function, and so provides a rich framework for biomedical research.

## Abstract 2

Using data from model organisms, the authors have generated a large-scale human protein-protein interaction map. The map can be used to predict the function of human proteins.

## Background

Physical interactions between proteins underpin most biological processes. For this reason, large-scale protein-interaction mapping projects have been initiated in several model organisms [1-6]. Unfortunately, projects of a similar scale have not yet been described for mammalian systems, with the result that our global understanding of protein function remains less advanced in mammals than in lower eukaryotes. However, many physical interactions are conserved between species, so it should be possible to infer information about human protein interactions and protein function using data from model organism protein-interaction datasets [7,8].

To transfer information on gene function between two genomes requires the identification of orthologous genes in the two genomes (that is, genes that are descended from a common ancestor and share biological functions). However, the identification of gene orthologs is often not a trivial problem; gene duplications can result in a single gene having multiple potential orthologs in a second species. In addition, it is necessary to distinguish true gene orthologs from 'out-paralogs' (that is, genes that arose from a gene-duplication event before the divergence of two species, and so are unlikely to share functions) [9]. One method that addresses both these problems is the InParanoid algorithm, which first identifies potential orthologs by best pairwise similarity searches, and then clusters these orthologs into groups of likely co-orthologs, with each ortholog assigned a score representing the confidence that it is the main ortholog [9]. We have used the orthology relationships identified by the InParanoid algorithm to construct a putative human protein-interaction map based solely on high-throughput interaction datasets from model organisms. We show that this approach successfully identifies functionally related human proteins, and so can be used to assign putative functions to many novel human genes. The resulting network provides a framework for human biology and acts as a guide for a future experimental human protein-interaction mapping project.

## Results

### Generation of a human protein-interaction map

**Figure 1:**
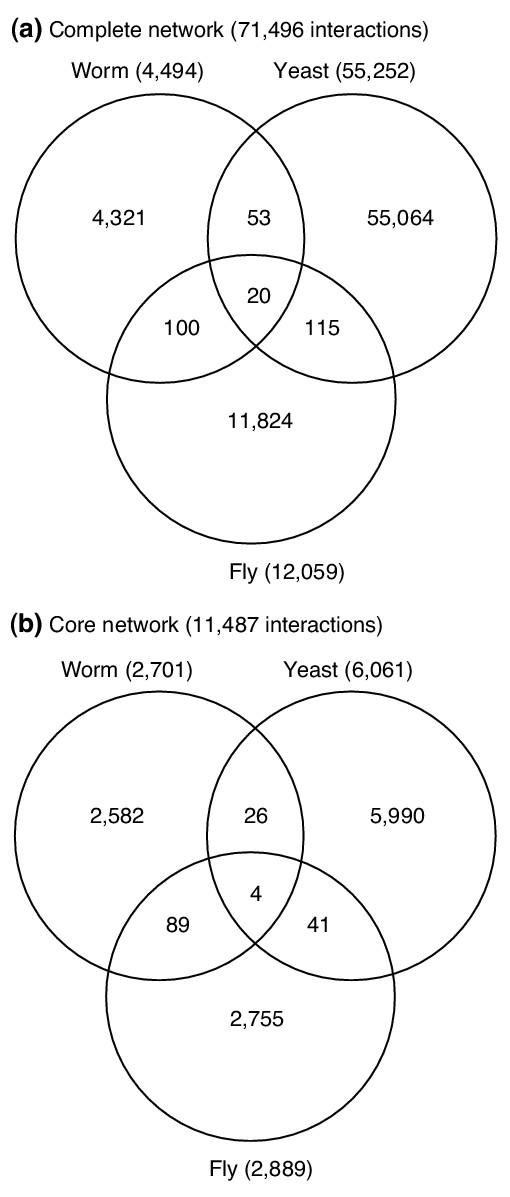
Sources of predicted human protein interactions. **(a) **The number of human protein interactions predicted by the interaction maps from each model organism. **(b) **The number of human protein interactions predicted by the core higher-confidence interactions from each organism. As explained in the text, core interactions are those that reconfirmed when retested (worm), or had an interaction score of greater than 0.5 (fly) or were identified more than once in a single assay (yeast, worm).

Protein interactions are often evolutionarily conserved between orthologous proteins from different species [7]. Hence we reasoned that a human protein-interaction map could be constructed using data from model organism protein-interaction mapping projects. We obtained the data from seven experimental and four computationally predicted protein-interaction maps from *Saccharomyces cerevisiae *[1-4,10,11], *Drosophila melanogaster *[5] and *Caenorhabditis elegans *[6]. For each interacting protein, we identified potential human orthologs using the InParanoid algorithm [9]. A human protein interaction is predicted if both interaction partners from a model organism have one or more human orthologs. Using this strategy, we were able to generate a human interaction network comprising 71,496 interactions between 6,231 human proteins. The sources of these predicted interactions are summarized in Table 1 and Figure 1a, and all the interactions are available in Additional data file 1 available online with this article and can also be searched or downloaded from our website [12].

### Assessment of the accuracy of the interaction datasets

**Figure 2:**
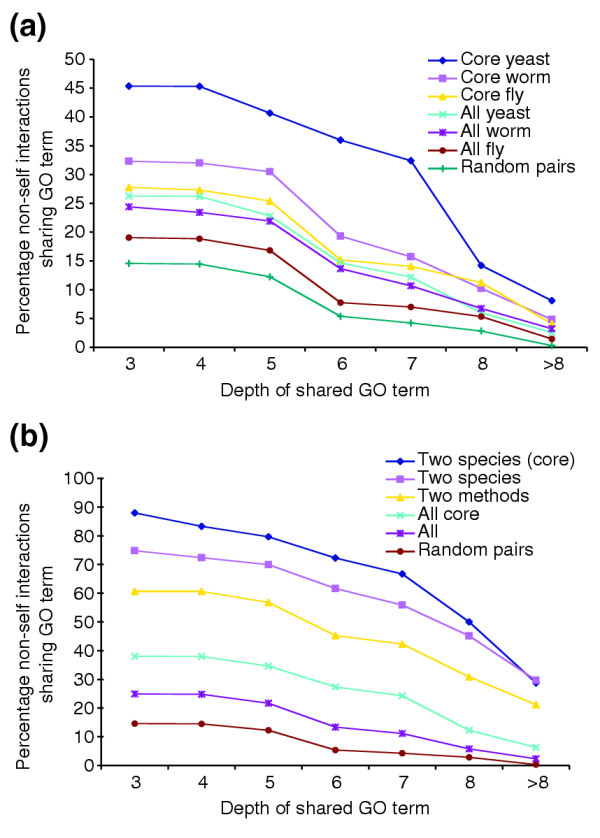
Filtering interaction datasets to improve their accuracy. **(a) **The percentages of interactions sharing GO terms at various depths in the GO hierarchy are compared for interactions predicted by the high-confidence interactions from each model organism (core yeast, core worm and core fly), as well as for the complete datasets from each organism (all yeast, all worm, all fly). For comparison, the percentage of shared GO terms is shown for a randomly generated network of the same size as the complete human network (random pairs). The *x*-axis indicates the depth in the GO hierarchy being considered, and the *y*-value the percentage of interaction partners (with known GO annotations) that share GO annotations at this depth or deeper. **(b) **The percentages of interactions sharing GO terms at different levels in the GO hierarchy are compared for interactions predicted by core interactions in two or more species (two species (core)), by interactions in the complete datasets of two or more species (two species), for interactions predicted by more than one experimental method in yeast (two methods), by any core interaction (all core), by any interaction (all), or by a randomly generated interaction network of the same size as the complete human interaction network (random pairs). All values shown are the percentage of non-self interactions between pairs of proteins that both have at least one associated GO term at the indicated depth in the GO hierarchy.

In the absence of a comprehensive set of verified human protein interactions, we required another method to assess the accuracy of the interaction network. Proteins that interact physiologically are expected to have related functions. Therefore high-quality interaction datasets should predict a greater proportion of interactions between functionally related proteins than low quality datasets. The functions of human proteins can be systematically described using the Gene Ontology (GO) annotations [13] available from Ensembl [14-17]. GO annotations provide a hierarchical description of gene functions with general functions described by GO annotations at the top levels of the hierarchy and very precise functions described by terms deeper in the hierarchy. Because physiologically interacting proteins are expected to have related, but non-identical functions, they are expected to share some, but not all GO annotations. Therefore, one method to evaluate an interaction dataset is to count the proportion of interactions that connect proteins that share common GO terms [5]. For the complete predicted human interaction network, 25% of interaction partners share at least one GO term, which is many more than observed with a randomly generated network of the same size (15% of interactions). To confirm that this result did not just apply to quite general GO annotations, we calculated the proportion of interaction partners that share GO annotations at depths 3 to 8 and greater than 8 in the GO hierarchy. We found that the predicted interaction network preferentially connects proteins that share GO annotations at any level of the GO hierarchy (see Figure 2). This suggests that the interaction network indeed preferentially connects functionally related human proteins.

We then used the same strategy to compare the accuracy of human interactions predicted by data from the three different model organisms. If the interactions from a particular model organism dataset predict fewer interactions between functionally related human proteins than the other datasets, then this dataset should be considered less reliable as a source of candidate human protein interactions. As shown in Table 1 and Figure 2a, interactions predicted by the complete yeast and worm datasets are slightly better at connecting functionally related human proteins than those predicted by the fly dataset, suggesting that these interactions can be considered with higher confidence. This result is especially interesting given that the yeast interaction map is an order of magnitude larger than the fly or worm maps, confirming that the fly and worm interaction maps currently have a relatively low coverage.

Next we asked how the confidence in the assignment of gene orthologs affects the accuracy of an interaction. For each predicted interaction, an orthology confidence score was calculated by summing the InParanoid orthology confidence scores for the two human and two model organism proteins (see Materials and methods). Of the predicted interactions, 24,897 have the maximum possible confidence score of 4. Of these interactions, 28%, 24% and 13% connect proteins that share GO terms at depths of 3, 5 or 7 in the GO hierarchy (excluding proteins without GO annotation). In contrast, for interactions with an orthology confidence score less than 4, these figures are 24%, 20% and 10%. Hence we conclude that the predicted human interactions with high-confidence orthology assignments can be considered more reliable than those interactions with less confidence in their orthology assignments. This confirms that the confidence scores assigned using InParanoid are indeed likely to be useful predictors of functional conservation.

### A core dataset of high-confidence protein interactions

The worm and fly interaction mapping projects both defined a subset of high-confidence 'core' interactions that have the greatest experimental support (Figure 1b). For the worm interaction map these were defined as interactions identified more than once, or that reconfirmed when retested in the two-hybrid assay [6]. In the fly interaction map each interaction has an associated confidence score, and interactions with a score greater than 0.5 are considered core interactions (the interaction score mainly depends upon the number of times each interaction was detected, the total number of interactions made by each protein and the local network clustering; see [5]). To generate a similar subset of yeast protein interactions, we defined core yeast protein interactions as those identified more than once by any single assay, consistent with previous analyses of the individual datasets [1-3,11]. As shown in Figure 2a and Table 1, for all three species these core interactions predict a greater proportion of human interactions that share GO terms than the total datasets. Indeed all three core interaction maps are of similar accuracy, so we combine their predicted interactions into a core network of 11,487 higher-confidence human protein interactions (summarized in Table 2 and available as Additional data file 2). Of these core interactions, 38%, 35% and 24% connect proteins that share GO terms at depths of 3, 5 or 7 in the GO hierarchy (excluding proteins with no GO annotations).

### Combining interaction datasets to generate high-confidence networks

It has been shown previously that protein interactions detected by more than one high-throughput interaction assay are more accurate [11]. We find that this is also true for human protein interactions predicted by yeast protein interactions detected by more than one method (see Figure 2b and Table 1). It has also been suggested that protein interactions are more likely to represent physiologically important interactions if they have been detected between orthologous protein pairs from two or more species [7,18]. To test this hypothesis we identified 288 human protein interactions predicted by interactions in two or more model organisms (Figure 1, Table 1). Remarkably, 75%, 70% and 56% of these interactions share GO terms at depths of 3, 5 or 7 in the GO hierarchy, respectively (Figure 2b). Indeed, for interactions derived from core interaction datasets, these figures rise to 88%, 80% and 67% of interactions. Hence, protein interactions predicted by data from multiple species can be considered with very high confidence.

### Using the interaction network to predict human gene function

Because physiologically interacting proteins often have similar functions (Figure 2), it should be possible to predict the functions of a novel human protein if it interacts with proteins of known function. To address how well our interaction map could be used for this purpose, we asked whether the known GO terms of a protein could be predicted using only the GO terms of its interaction partners. As shown in Table 3, GO terms associated with at least one of a gene's core interaction partners predict GO terms associated with that gene with an accuracy of around 8%. However, GO terms associated with at least two, three, four or five of a gene's interaction partners have 22%, 30%, 37%, 42% and 45% probabilities, respectively, of also being associated with that gene (Table 3). Although these values may vary for different GO terms, as shown in Additional data file 3, the accuracy and coverage of these GO term predictions are very similar for GO terms at different levels in the GO hierarchy, and so can be used as an approximate indication of the confidence in a prediction of gene function. Hence the network can be used to predict GO terms for a human gene of unknown function, with the approximate confidence in the GO prediction determined by the number of interaction partners that share the GO term.

The ability to provide a reasonably accurate prediction of a gene's GO terms means that we can use the interaction network to provide probabilistic gene function predictions for novel human proteins and also to predict additional functions for proteins with some known functions. The core interaction map contains 864 proteins with no functional annotations. About 10% of these proteins interact with two or more proteins that share GO terms. The probabilistic predictions of the functions of these novel proteins are listed in Additional data file 4. Often these predicted functions are also supported by the known functions of the protein domains predicted to be encoded by these novel genes (see Additional data file 4). For example, ENSG00000028310 encodes a bromodomain and interacts with six proteins annotated as 'GO:0006355 regulation of transcription, DNA-dependent', ENSG00000080608 encodes an RNA-binding domain and interacts with five proteins annotated as 'GO:0006364 rRNA processing', and ENSG00000104863 encodes a PDZ domain and interacts with three proteins with the annotations 'GO:0005887 integral to plasma membrane, GO:0007242 intracellular signaling cascade' (Additional data file 4). The complete and core interaction maps also predict interactions for 448 and 292 human disease genes (listed in Additional data file 5), of which 55 interact with two or more proteins in the core interaction network that share a GO annotation. The functional predictions for these 55 genes are listed in Additional data file 6.

## Discussion

### A framework for human biology

We report here the use of data from model organism protein-interaction mapping projects to predict a network of human protein interactions. This network consists of over 70,000 interactions that connect over one-third of all the predicted human proteins, including 1,482 proteins of unknown function and 448 proteins encoded by human disease genes. The physiological relevance of this network is supported by its ability to preferentially connect human proteins that share biological functions (Figure 2). Indeed the network can be successfully used to predict the functions of a gene using the known functions of its interaction partners (Table 3). As such, the network should provide a rich source of functional hypotheses for researchers interested in the functions of one or many human proteins.

The accuracy and coverage of the interactions predicted in this network depend primarily on two parameters: the quality of the original model organism interaction datasets; and the ability to identify the human orthologs of a model organism protein. Our analysis suggests that the raw yeast and worm protein-interaction datasets are currently slightly more accurate than the raw fly interaction dataset, but that when filtered for high-confidence interactions the three interaction maps are of very similar accuracy (see Table 1 and Figure 2). The fly and worm interaction maps both have a much lower coverage than the yeast interaction network, most probably because they both only represent the results of a single interaction-mapping project. The continuation of these model organism protein-interaction mapping projects to generate higher coverage interaction maps will greatly enhance our ability to predict human protein interactions.

For the identification of gene orthologs, we used the InParanoid algorithm. InParanoid offers several important benefits compared to simple 'reciprocal best hit' sequence-similarity searches [9]. First, many genes from lower eukaryotes have multiple co-orthologs in humans, which can be identified using InParanoid, but not by simple one-to-one sequence-similarity searches. Second, InParanoid can successfully distinguish these true co-orthologs from paralogs that arose before a speciation event (which are unlikely to retain similar functions). Finally, each potential ortholog in a group of co-orthologs identified by InParanoid has an associated score that represents the likelihood that it is the main ortholog of a gene. We have summed these confidence scores to provide an orthology confidence score for each predicted human protein interaction in our network. These high-confidence ortholog interactions connect a greater proportion of functionally related human proteins, suggesting that the InParanoid confidence score is indeed a useful tool for predicting the likely physiological relevance of a predicted protein interaction.

The ability to successfully predict human protein functions using the results of model organism protein-interaction mapping projects highlights both the relevance of model organism protein-interaction mapping projects to understanding human biology and also the benefits that would result from an experimental human protein-interaction mapping project. Although the interaction network can currently accurately predict only a subset of the known functions of a gene, this should improve as more protein-interaction data becomes available. For this reason, we strongly encourage the continuation of model organism protein-interaction mapping projects.

### Methods of verifying protein-interaction datasets

We also assessed the relative merits of three different methods to improve the accuracy of protein-interaction maps. The first strategy is to define a subset of interactions detected more than once with a single assay [1-3,6]. We found that this approach leads to an approximately 1.5- to 2.7-fold increase in the proportion of predicted human interactions that share GO terms (Figure 2b). The second strategy is to define a subset of interactions that have been identified by more than one interaction assay. This results in around a 2.3- to 8-fold improvement in the prediction of associations between proteins that share GO terms (Figure 2b). The final strategy is to define a subset of interactions that are predicted by interactions from more than one model organism, which results in around a 3- to 12-fold improvement in the proportion of interactions between proteins sharing GO terms (Figure 2b).

With all these filtering methods, the greatest improvements are seen when considering the proportion of interactions that share GO terms deep within the GO hierarchy; that is, the filtering steps dramatically improve the proportion of interactions between proteins with very closely related functions. We conclude that using interaction data derived from a second interaction assay or from a second species both represent excellent methods to improve the accuracy of protein-interaction maps. Because of the small number of protein-interaction assays that have been adapted to a high-throughput format, we suggest that constructing a second interaction map in a related organism using the same assay may be an efficient way to produce a high-confidence interaction map. This strategy is somewhat similar to using phylogenetic footprinting to identify functional noncoding DNA, so we suggest it should be named 'interaction footprinting'. Using the relatively low-coverage model organism interaction datasets currently available, only a small proportion of interactions can be verified by interaction footprinting. The continuation of these model organism interaction mapping projects will not only provide a much richer framework of predicted human protein interactions, but will also allow many more interactions to be verified using the interaction footprinting strategy. However, such an approach will be limited to providing information on those proteins and interactions that are conserved between vertebrates and invertebrates.

### Strategies for completing the human interaction map

The interactions described here provide a first-draft human protein-interaction map that can be used to predict interactions and functions for genes of interest to a particular researcher. However, the map also provides a framework from which a complete human protein-interaction map could be generated. Firstly, the map could be used to identify subsets of high-confidence, evolutionarily conserved interactions from the results of large- or medium-scale human interaction-mapping projects. For example the map verifies 51 of 296 yeast two-hybrid interactions detected for human proteins involved in mRNA decay [19]. Alternatively, the interactions predicted here could be directly experimentally validated using an assay that allows rapid testing of binary interactions (such as the yeast or mammalian two-hybrid assays [20] or protein fragment complementation assays [21]). This would represent a cost-effective strategy to produce a high-confidence human protein-interaction map because it massively reduces the number of candidate interactions that need to be tested. Finally, the map identifies 17,300 (23,531 - 6,231) human genes for which no protein interactions are predicted from model organism interaction datasets. Many of these proteins are likely to be vertebrate- or mammalian-specific, and are the most logical choices for bait proteins for the discovery phase of an experimental human protein-interaction mapping project.

## Materials and methods

### Model organism protein-interaction datasets

The interaction datasets used to generate the draft human protein-interaction network were two-hybrid-based interaction maps for *D. melanogaster *[5] and *C. elegans *[6] and a list of *S. cerevisiae *protein-interactions compiled by Von Mering *et al. *[11] from two two-hybrid [1,2], two complex purification [3,4], one genetic [10], and four *in silico*-predicted interaction datasets (which used correlated mRNA expressions, conserved gene neighbourhood, gene co-occurrence or gene fusion events to predict protein interactions [11]). Table 4 shows the number of unique interactions in each dataset, the methods used to generate each dataset, and the URLs from which the datasets were obtained.

### Identification of gene orthologs and construction of the interaction network

The human orthologs of yeast, worm and fly genes were identified using the InParanoid algorithm, which is designed to distinguish true orthologs from out-paralogs that arose from gene duplications before the divergence of two species [9]. The InParanoid algorithm first identifies potential orthologs by best pairwise similarity searches, and then clusters these orthologs into groups of probable co-orthologs, with each ortholog assigned a score representing the confidence that it is the main ortholog. For each interaction data source, we obtained SWISS-PROT/TrEMBL accessions for each interacting protein using the Ensmart data-mining tool [16,17] (for worm and fly genes) or both SWISS-PROT [22] and a TrEMBL conversion file kindly provided by Paul Kersey, EBI, Hinxton, UK (for yeast genes). Potential human orthologs of these genes were then identified using the pre-computed InParanoid results (version 2.3, available from [23]), and the results converted to nonredundant Ensembl (v19.34a.1, genome assembly NCBI34) gene IDs using Ensmart (v19.1) 1 [16,17]. In total, InParanoid identifies 9,500 human genes with at least one ortholog in at least one of worm, fly or yeast. For each potential ortholog in a group of co-orthologs, the InParanoid algorithm calculates a score that represents the confidence that it is the main ortholog. In this scoring system, the main ortholog always receives a score of 1, with the other co-orthologs receiving scores ranging between 0 and 1, calculated according to their similarity to the main ortholog [9]. As an indication of the confidence we have in the orthology relationships between a pair of interacting proteins from a model organism and a predicted pair of interacting human proteins, we calculate a confidence score by summing the InParanoid confidence scores for each of the four proteins. Hence, each interaction has an associated score ranging from 0 to 4 that represents the confidence that both human proteins represent the main orthologs of the model organism proteins, and vice versa.

Core interactions were defined as those predicted by worm interactions identified more than once or that reconfirmed when retested in the two-hybrid assay [6], by fly interactions with an interaction score greater than 0.5 [5], or by yeast interactions detected two or more times by a single assay [1-3,11].

### Assessment of the interaction data

Human GOs (at levels 3 or deeper in the GO hierarchy) were obtained from Ensembl (v19.34a.1) [14,15] using Ensmart (v19.1) [16,17]. The GO terms 'unknown molecular function/biological process/cellular compartment' were discarded in all subsequent analyses. To validate the accuracy of the interaction data, we calculated the percentage of interactions that shared at least one GO term. To confirm that the results did not just apply to very general GO annotations, we calculated the proportion of interacting proteins that shared a GO annotation at levels 3 to 8 and greater than 8 in the GO hierarchy. For all of these analyses we ignored proteins with no associated GO annotations. Moreover, self-interactions were excluded because they will always share GO terms and so bias the results.

### Prediction of gene functions

To predict the GO terms of a protein, we identified all the GO terms associated with *x *or more of its interaction partners (where *x *varied from 1 to 6). To validate the accuracy and coverage of this approach we predicted GO terms for genes that already have associated GO terms. The accuracy was calculated as the total number of correct GO term predictions divided by the total number of GO term predictions. The coverage was calculated as the total number of correct GO term predictions divided by the total number of known GO terms. This analysis was repeated, but only considering individually GO terms at depths of 3 to 8 and greater than 8 in the GO hierarchy (see Additional data file 3). To avoid biasing the results we again ignored self-interactions. For the same reason, we also only counted once GO terms associated with more than one interaction partner predicted by the same source interaction from a model organism. The InterPro protein domains [24] encoded by each human gene were obtained from Ensembl using Ensmart. Genes of unknown function were defined as those having no associated GO terms, and disease genes were as defined by Ensembl using the Online Mendelian Inheritance in Man (OMIM) database as a reference [25].

## Additional data files

The following additional data files are available with the online version of this article: Additional data file 1 contains a complete list of predicted human protein interactions; this dataset contains every human protein interaction that is predicted by a protein interaction from any of seven experimental and four computationally-predicted protein interaction maps from *Saccharomyces cerevisiae *[1-4,10,11], *Drosophila melanogaster *[5] and *Caenorhabditis elegans *[6].

Additional data file 2 contains a list of all core human protein interactions. This represents a subset of high-confidence human protein interactions that is predicted by model organism protein interactions with greater experimental support. In the worm interaction map, these are defined as interactions that reconfirmed when retested in the Y2H assay [6]. In the fly interaction map, each interaction has an associated confidence score, and interactions with a score greater than 0.5 are considered core interactions (the interaction score mainly depends upon the number of times each interaction was detected, the total number of interactions made by each protein and the local network clustering [5]). To generate a similar subset of yeast protein interactions, we defined core yeast protein interactions as those identified more than once by any single assay. Each entry in the core and complete interaction networks contains the following tab delimited information: Gene 1 Id, Ensembl gene ID for human interaction partner 1; Gene 1 description, alternative names for human Gene 1 (from Ensembl); Gene 2 Id, Ensembl gene ID for human interaction partner 2; Gene 2 description, alternative names for human Gene 2 (from Ensembl); Source Organism, the model organism protein interaction dataset that predicts this human protein interaction; Ortholog 1, model organism interaction partner 1 from the model organism protein interaction that predicts the human protein interaction; Ortholog 2, model organism interaction partner 2 from the model organism protein interaction that predicts the human protein interaction; and Ortholog score, a confidence score for the human protein interaction based on the likelihood that the two human proteins are the functional orthologs of the two model organism proteins. The score ranges from 0 (no confidence) to 4 (high confidence). The score is calculated as the sum of the InParanoid confidence scores for each gene orthology assignment. A score of 4 means that both of the human genes and both of the model organism genes are all the main orthologs in their groups of co-orthologs according to InParanoid. These represent higher confidence human protein interactions. Description, this field contains the original annotation for the model organism protein interaction; for worm interactions this indicates whether the interaction is in the core dataset of interactions found more than once (CORE_1), or interactions that reconfirmed when retested (CORE_2), or non-core interactions that did not reconfirm (NON_CORE) [6]. For fly interactions this indicates the interaction score. This score mainly depends upon the number of times each interaction was detected, the total number of interactions made by each protein and the local network clustering, see [5] for details. A score >0.5 is considered high confidence. For yeast protein interactions, these are the annotations of von Mering *et al*. [11] and contain the following information: experimental/computation method (and the number of times the interaction was detected); Von Mering *et al*.'s confidence assignment; and whether the interaction was previously known in the literature. For more information, please see [11].

Additional data file 3 lists the accuracy and coverage of GO term predictions at different levels in the GO hierarchy; Additional data file 4 lists gene function predictions for 85 human genes of unknown function; Additional data file 5 lists human disease genes with predicted protein interactions; and Additional data file 6 lists gene function predictions for 55 human disease genes.

## Supplementary Material

Additional data file 1 A complete list of predicted human protein interactions Click here for additional data file

Additional data file 2 A list of all core human protein interactions Click here for additional data file

Additional data file 3 The accuracy and coverage of GO term predictions at different levels in the GO hierarchy Click here for additional data file

Additional data file 4 Gene function predictions for 85 human genes of unknown function Click here for additional data file

Additional data file 5 Human disease genes with predicted protein interactions Click here for additional data file

Additional data file 6 Gene function predictions for 55 human disease genes Click here for additional data file

## Figures and Tables

**Table 1:** The number and accuracy of human protein interactions predicted by different model organism protein-interaction datasets

Data source	Predicted human interactions	Interactions sharing GO terms	
		
		Number	%
All	71,496	12,724	24.9
Yeast	55,231	10,727	26.2
Fly	12,059	1,404	19.0
Worm	4,494	753	24.4
All core	11,487	3,133	38.1
Core yeast	6,061	2,146	45.4
Core fly	2,889	488	27.8
Core worm	2,701	597	32.3
Two species	288	154	74.8
Two species (core)	160	95	88.0
Two methods	2,166	829	60.6
Random pairs	71,496	6,053	14.6

The table lists the total number of interactions predicted by each interaction dataset, and the number of these interactions that connect proteins that share at least one GO term (at level 3 or deeper in the GO hierarchy). The percentages are relative to the total number of non-self interactions where both proteins have at least one GO annotation. All, all predicted human protein interactions; Yeast/worm/fly, interactions predicted by the yeast, worm or fly interaction maps; All core, all interactions predicted by the high-confidence subsets of each model organism interaction map (see Materials and methods); Two species, interactions predicted by more than one model organism interaction map; Two species (core), interactions predicted by the high-confidence subset of interactions from more than one model organism; Two methods, interactions predicted by data derived from more than one different interaction assay; Random pairs, the data for a randomly generated interaction network.

**Table 2:** The number of interactions, genes, novel genes and disease genes in the complete and core human interaction networks

Network	Interactions	Genes	Novel genes	Disease genes
Complete	71,496	6,231	1,482	448
Core	11,487	3,872	864	292

The complete network consists of all human protein interactions predicted by model organism protein-interaction datasets. The core network consists of all the human interactions predicted by the high-confidence subsets of each interaction network (see Materials and methods). Novel genes are defined as those without GO annotations. Disease genes are defined by the OMIM database [25], available from Ensembl [16].

**Table 3:** The approximate accuracy and coverage of GO terms predicted by the core and complete interaction networks

Number of interactors with GO term	Core data		Complete data	
		
	Accuracy	Coverage	Accuracy	Coverage
1+	8	26	3	35
2+	22	11	8	19
3+	30	7	11	14
4+	36	5	15	11
5+	42	4	18	8
6+	45	3	20	7

The approximate accuracy and coverage of GO term predictions were calculated for every gene in the core or complete interaction networks with at least one known GO term. The GO terms of a gene are predicted using the GO terms of any of its interaction partners (1+), or GO terms shared by at least two to six of its interaction partners (2+ to 6+). Accuracy is calculated as the number of correctly predicted GO terms divided by the total number of predicted GO terms. Coverage is calculated as the number of correctly predicted GO terms divided by the total number of known GO terms associated with each gene. These values are similar for GO annotations at different levels of the GO hierarchy (see Additional data file 3).

**Table 4:** Sources of model organism protein-interaction data

Dataset	Interactions	Type	Reference	URL
Fly	20,020	Two-hybrid	[5]	[26]
Worm	4,605	Two-hybrid	[6]	[27]
Yeast	78,391	Total	[11]	[11]
	5,125	Two-hybrid		
	49,313	Complex purification		
	886	Genetic		
	23,844 (23,399)	*In silico *(*In silico *only)		

The table lists the total number of interactions contained in each model organism dataset, together with the method used to identify interactions, the publication reference, and the website (URL) from which the interaction dataset was obtained. For each dataset, the non-redundant number of unique interactions between unambiguously identified proteins is shown. For the yeast interactions, the total number of interactions is shown, as well as the number of interactions identified using each detection method. *In silico *only are interactions only predicted by *in silico *methods without any confirmation from the experimental datasets.
