# Supplementary material for: A first-draft human protein-interaction map
Source: Genome Biol. 2004 Aug 13;5(9):R63. doi: 10.1186/gb-2004-5-9-r63 (PMC522870; doi:10.1186/gb-2004-5-9-r63)
Supplement: Additional data file 3 — The accuracy and coverage of GO term predictions at different levels in the GO hierarchy [file gb-2004-5-9-r63-s3.pdf]

Lehner & Fraser Supplementary figure 1 (page 1 of 3 pages)

The accuracy and coverage of GO term prediction are similar for GO terms at different depths in the GO hierarchy. To predict the GO terms of a protein, we identified all of the GO terms associated with *x* or more of its interaction partners in the core interaction network (where *x* varied from 1 to 6). This was done for GO terms found at maximum depths of 3 to 8 or >8 in the GO hierarchy. To validate the accuracy and coverage of this approach we predicted GO terms for every gene that already has at least one associated GO term. **(A)** Accuracy of GO term predictions for GO terms at different levels in the GO hierarchy. The proportion of correctly predicted GO terms are shown for GO terms shared by 1 to 6 of a protein's interaction partners. Accuracy was calculated as the total number of correct GO term predictions divided by the total number of GO term predictions. **(B)** The coverage of GO term predictions is calculated as the total number of correct GO term predictions divided by the total number of known GO terms for the same GO predictions as in (A). **(C)** The total number of GO terms at each depth in the GO hierarchy for every protein in the core interaction network with at least one associated GO term. **(D)** The total number of GO terms predicted by x or more core interaction partners at each depth in the GO hierarchy for every protein in the core interaction network with at least one associated GO term. **(E)** The number of correctly predicted GO terms predicted by x or more core interaction partners at each depth in the GO hierarchy for every protein in the core interaction network with at least one associated GO term. To avoid biasing the results we ignored self-interactions, as these will always share GO terms. For the same reason, we also only counted once GO terms associated with more than one interaction partner predicted by the same source interaction from a model organism.

Lehner & Fraser Supplementary figure 1 (page 2 of 3 pages)

A

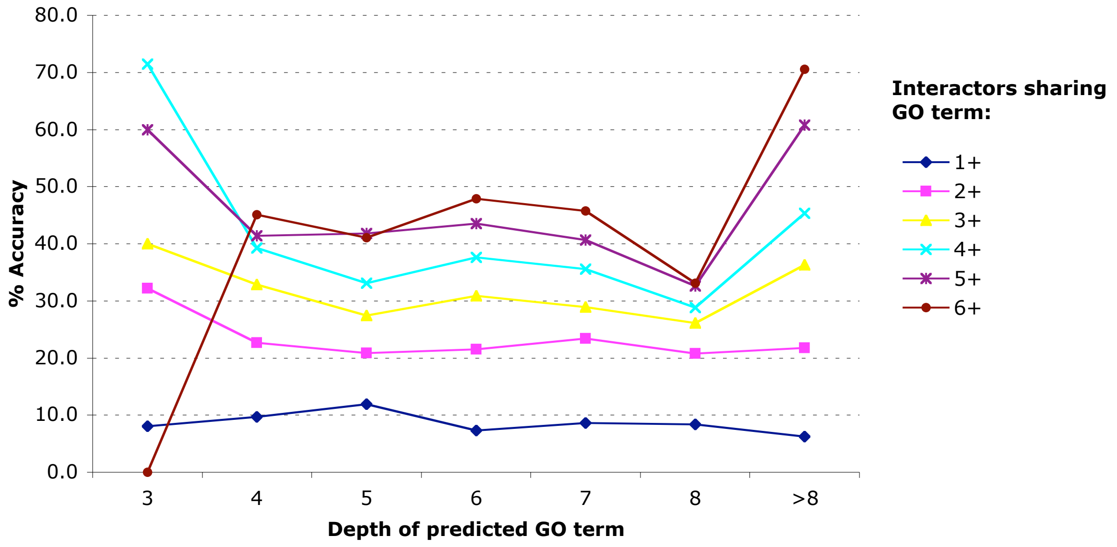

B

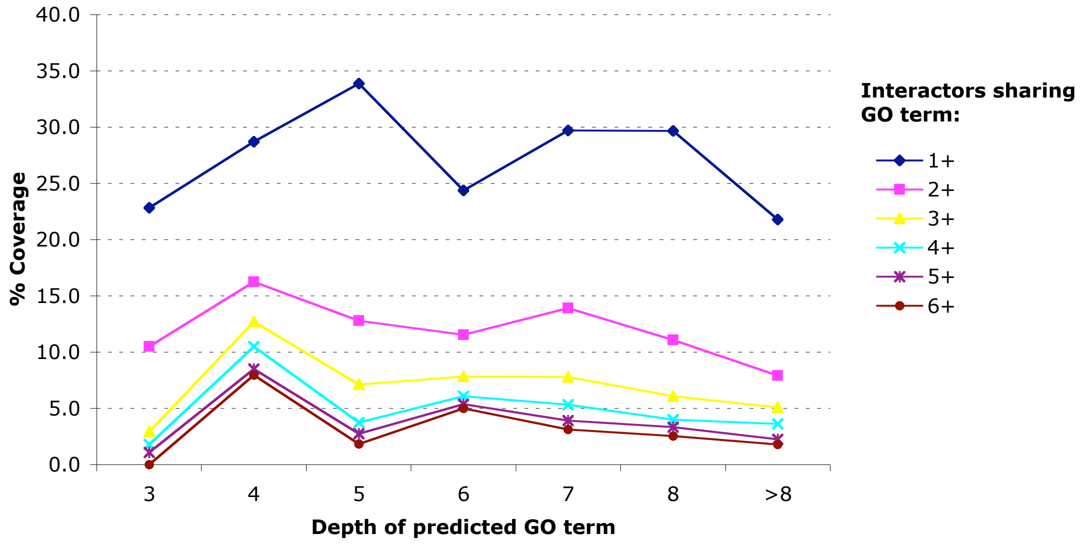

Lehner & Fraser Supplementary figure 1 (page 3 of 3 pages)

C

Total number of annotated GO terms.

	GO depth							
	3	*4*	*5*	*6*	*7*	*8*	*>8*	*All*
	779	6660	7119	6071	6523	6641	7051	40844

D

Total number of GO terms predicted by x or more core interaction partners.

	GO depth							
	3	*4*	*5*	*6*	*7*	*8*	*>8*	*All*
X=1	779	6660	7119	6071	6523	6641	7051	40844
X=2	90	1614	1536	982	1125	999	736	7082
X=3	20	871	652	463	508	437	284	3235
X=4	7	601	284	295	284	260	161	1892
X=5	5	461	165	225	182	193	74	1305
X=6	1	397	112	190	129	145	51	1025

E

Number of correctly predicted GO terms predicted by x or more core interaction partners.

	GO depth							
	3	*4*	*5*	*6*	*7*	*8*	*>8*	*All*
X=1	63	646	849	445	562	558	440	3563
X=2	29	366	321	211	263	208	160	1558
X=3	8	286	179	143	147	114	103	980
x=4	5	236	94	111	101	75	73	695
x=5	3	191	69	98	74	63	45	543
x=6	0	179	46	91	59	48	36	459
