# Supplementary material for: A first-draft human protein-interaction map
Source: Genome Biol. 2004 Aug 13;5(9):R63. doi: 10.1186/gb-2004-5-9-r63 (PMC522870; doi:10.1186/gb-2004-5-9-r63)
Supplement: Additional data file 4 — Gene function predictions for 85 human genes of unknown function [file gb-2004-5-9-r63-s4.doc]

**Lehner and Fraser Supplementary table 3.**

Gene function predictions for 85 human genes of unknown function. The table lists all the genes from the core interaction dataset that have no associated GO terms themselves, but have 2 or more interaction partners that share a common GO term. All of these GO terms and their accessions are listed. The number of interaction partners associated with each GO term is indicated in brackets for each GO term. The GO predictions are classified according to the approximate probability that the predicted GO term will be associated with the novel gene (see table 3). The predicted Interpro protein domains encoded by the gene are shown for comparison, and in many cases strongly support the predicted functions.

|  |  |  | Predicted GO terms |  |  |
| --- | --- | --- | --- | --- | --- |
| Novel gene | Interpro domains | > 40% probability | 37% probability | 30% probability | 22% probability |
| ENSG00000007392.4 | IPR004882 Protein of unknown function DUF259, IPR004882 Protein of unknown function DUF259 |  | GO:0005681 (4) spliceosome complex, GO:0006371 (4) mRNA splicing | GO:0005634 (3) nucleus, GO:0005732 (3) small nucleolar ribonucleoprotein complex, GO:0008248 (3) pre-mRNA splicing factor activity | GO:0000245 (2) spliceosome assembly, GO:0003723 (2) RNA binding |
| ENSG00000028310.3 | IPR001487 Bromodomain, IPR001472 Bipartite nuclear localization signal | GO:0005634 (6) nucleus, GO:0006355 (6) regulation of transcription, DNA-dependent | GO:0005669 (4) transcription factor TFIID complex | GO:0006352 (3) transcription initiation, GO:0016251 (3) general RNA polymerase II transcription factor activity | GO:0003677 (2) DNA binding, GO:0005515 (2) protein binding, GO:0006366 (2) transcription from Pol II promoter |
| ENSG00000047932.2 | IPR000408 Regulator of chromosome condensation, RCC1, IPR001478 PDZ/DHR/GLGF domain | GO:0005634 (5) nucleus |  |  | GO:0006355 (2) regulation of transcription, DNA-dependent, GO:0016787 (2) hydrolase activity |
| ENSG00000052749.3 | IPR001687 ATP/GTP-binding site motif A (P-loop), IPR001687 ATP/GTP-binding site motif A (P-loop) | GO:0005634 (5) nucleus, GO:0006364 (5) rRNA processing |  | GO:0005524 (3) ATP binding, GO:0005730 (3) nucleolus | GO:0003676 (2) nucleic acid binding, GO:0004004 (2) ATP dependent RNA helicase activity, GO:0016787 (2) hydrolase activity |
| ENSG00000080608.3 | IPR001313 Pumilio/Puf RNA-binding | GO:0005634 (12) nucleus, GO:0006364 (5) rRNA processing | GO:0005524 (4) ATP binding, GO:0005730 (4) nucleolus | GO:0004004 (3) ATP dependent RNA helicase activity, GO:0016787 (3) hydrolase activity | GO:0003676 (2) nucleic acid binding, GO:0003677 (2) DNA binding, GO:0005871 (2) kinesin complex |
| ENSG00000087269.3 | IPR007276 Nop14-like protein, IPR001472 Bipartite nuclear localization signal |  | GO:0005634 (4) nucleus | GO:0006364 (3) rRNA processing | GO:0005737 (2) cytoplasm, GO:0006118 (2) electron transport |
| ENSG00000101997.1 |  |  |  |  | GO:0005070 (2) SH3/SH2 adaptor protein activity, GO:0005737 (2) cytoplasm, GO:0007267 (2) cell-cell signaling |
| ENSG00000103253.2 | IPR001279 Beta-lactamase-like | GO:0008152 (7) metabolism | GO:0005739 (4) mitochondrion | GO:0003824 (3) catalytic activity, GO:0016021 (3) integral to membrane | GO:0005215 (2) transporter activity, GO:0005634 (2) nucleus, GO:0006085 (2) acetyl-CoA biosynthesis, GO:0006810 (2) transport, GO:0008415 (2) acyltransferase activity, GO:0016740 (2) transferase activity, GO:0016787 (2) hydrolase activity |
| ENSG00000104863.1 | IPR001478 PDZ/DHR/GLGF domain, IPR004172 L27 domain |  |  | GO:0005887 (3) integral to plasma membrane, GO:0007242 (3) intracellular signaling cascade, GO:0016020 (3) membrane | GO:0005516 (2) calmodulin binding, GO:0005524 (2) ATP binding, GO:0005856 (2) cytoskeleton, GO:0006468 (2) protein amino acid phosphorylation, GO:0015629 (2) actin cytoskeleton, GO:0016740 (2) transferase activity |
| ENSG00000105185.1 | IPR002836 DNA-binding TFAR19-related protein |  | GO:0003735 (4) structural constituent of ribosome, GO:0005622 (4) intracellular, GO:0005840 (4) ribosome, GO:0006412 (4) protein biosynthesis | GO:0005842 (3) cytosolic large ribosomal subunit (sensu Eukarya) | GO:0005634 (2) nucleus |
| ENSG00000105618.3 | IPR002687 Pre-mRNA processing ribonucleoprotein, binding region, IPR002687 Pre-mRNA processing ribonucleoprotein, binding region | GO:0006371 (8) mRNA splicing, GO:0005681 (6) spliceosome complex, GO:0008248 (5) pre-mRNA splicing factor activity |  | GO:0005634 (3) nucleus, GO:0030529 (3) ribonucleoprotein complex | GO:0030532 (2) small nuclear ribonucleoprotein complex |
| ENSG00000106344.1 | IPR000504 RNA-binding region RNP-1 (RNA recognition motif), IPR001472 Bipartite nuclear localization signal | GO:0005634 (6) nucleus | GO:0005524 (4) ATP binding | GO:0004004 (3) ATP dependent RNA helicase activity, GO:0005730 (3) nucleolus, GO:0006364 (3) rRNA processing, GO:0016787 (3) hydrolase activity | GO:0003676 (2) nucleic acid binding |
| ENSG00000108671.2 | IPR000717 Proteasome component region PCI | GO:0005837 (12) 26S proteasome, GO:0005829 (8) cytosol, GO:0005524 (5) ATP binding, GO:0005634 (5) nucleus, GO:0016787 (5) hydrolase activity | GO:0005838 (4) proteasome regulatory particle (sensu Eukarya) | GO:0006508 (3) proteolysis and peptidolysis, GO:0008575 (3) proteasome ATPase activity |  |
| ENSG00000108963.3 | IPR002728 Diphthamide synthesis DPH2 protein, IPR005645 Protein of unknown function DUF341 |  |  | GO:0005634 (3) nucleus | GO:0003899 (2) DNA-directed RNA polymerase activity, GO:0003900 (2) DNA-directed RNA polymerase I activity, GO:0003901 (2) DNA-directed RNA polymerase II activity, GO:0003902 (2) DNA-directed RNA polymerase III activity, GO:0006350 (2) transcription, GO:0008283 (2) cell proliferation, GO:0016740 (2) transferase activity |
| ENSG00000110060.1 | IPR001406 tRNA pseudouridine synthase | GO:0006412 (16) protein biosynthesis, GO:0005622 (15) intracellular, GO:0005840 (9) ribosome, GO:0003735 (8) structural constituent of ribosome, GO:0005842 (6) cytosolic large ribosomal subunit (sensu Eukarya) | GO:0005843 (4) cytosolic small ribosomal subunit (sensu Eukarya) | GO:0005739 (3) mitochondrion, GO:0016740 (3) transferase activity | GO:0003677 (2) DNA binding, GO:0003899 (2) DNA-directed RNA polymerase activity, GO:0003900 (2) DNA-directed RNA polymerase I activity, GO:0003901 (2) DNA-directed RNA polymerase II activity, GO:0003902 (2) DNA-directed RNA polymerase III activity, GO:0005634 (2) nucleus, GO:0005730 (2) nucleolus, GO:0005762 (2) mitochondrial large ribosomal subunit, GO:0006350 (2) transcription, GO:0015934 (2) large ribosomal subunit, GO:0016020 (2) membrane, GO:0016021 (2) integral to membrane |
| ENSG00000110107.1 | IPR001680 G-protein beta WD-40 repeat, IPR003613 Zn-finger, modified RING | GO:0005634 (7) nucleus, GO:0006371 (5) mRNA splicing | GO:0005681 (4) spliceosome complex |  | GO:0003676 (2) nucleic acid binding, GO:0030532 (2) small nuclear ribonucleoprotein complex |
| ENSG00000110844.1 | IPR001202 WW/Rsp5/WWP domain, IPR002965 Proline-rich extensin, IPR002713 FF domain, IPR000694 Proline-rich region |  | GO:0005634 (4) nucleus, GO:0005681 (4) spliceosome complex, GO:0006371 (4) mRNA splicing | GO:0005732 (3) small nucleolar ribonucleoprotein complex, GO:0008248 (3) pre-mRNA splicing factor activity | GO:0000245 (2) spliceosome assembly, GO:0006357 (2) regulation of transcription from Pol II promoter |
| ENSG00000113598.1 | IPR001440 TPR repeat |  | GO:0016021 (4) integral to membrane |  | GO:0006886 (2) intracellular protein transport, GO:0007155 (2) cell adhesion |
| ENSG00000114107.1 |  |  |  |  | GO:0000059 (2) protein-nucleus import, docking, GO:0005643 (2) nuclear pore, GO:0005654 (2) nucleoplasm |
| ENSG00000115145.3 | IPR001452 SH3 domain, IPR000108 Neutrophil cytosol factor 2, IPR002014 VHS, IPR003903 Ubiquitin interacting motif |  |  | GO:0005634 (3) nucleus, GO:0007165 (3) signal transduction | GO:0005624 (2) membrane fraction |
| ENSG00000115761.2 | IPR001472 Bipartite nuclear localization signal, IPR001472 Bipartite nuclear localization signal | GO:0005634 (6) nucleus, GO:0006364 (5) rRNA processing |  |  | GO:0003676 (2) nucleic acid binding, GO:0005524 (2) ATP binding, GO:0008026 (2) ATP dependent helicase activity |
| ENSG00000115806.2 | IPR007583 GRASP55/65 |  |  |  | GO:0004299 (2) proteasome endopeptidase activity, GO:0005829 (2) cytosol, GO:0005837 (2) 26S proteasome, GO:0005839 (2) proteasome core complex (sensu Eukarya), GO:0006511 (2) ubiquitin-dependent protein catabolism, GO:0007049 (2) cell cycle |
| ENSG00000119285.2 |  | GO:0005634 (6) nucleus, GO:0006364 (6) rRNA processing |  |  | GO:0003676 (2) nucleic acid binding, GO:0005524 (2) ATP binding, GO:0005622 (2) intracellular, GO:0005730 (2) nucleolus, GO:0005871 (2) kinesin complex, GO:0016787 (2) hydrolase activity |
| ENSG00000119946.1 | IPR000644 CBS domain |  |  | GO:0005524 (3) ATP binding | GO:0005737 (2) cytoplasm, GO:0005739 (2) mitochondrion, GO:0006434 (2) seryl-tRNA aminoacylation, GO:0016740 (2) transferase activity, GO:0016874 (2) ligase activity |
| ENSG00000120800.1 | IPR001687 ATP/GTP-binding site motif A (P-loop) | GO:0005634 (11) nucleus, GO:0006364 (6) rRNA processing |  | GO:0003676 (3) nucleic acid binding, GO:0005524 (3) ATP binding, GO:0005622 (3) intracellular, GO:0005730 (3) nucleolus | GO:0005739 (2) mitochondrion, GO:0005840 (2) ribosome, GO:0005871 (2) kinesin complex, GO:0006412 (2) protein biosynthesis |
| ENSG00000121022.4 | IPR000555 Mov34 family, IPR000555 Mov34 family | GO:0005634 (5) nucleus | GO:0005737 (4) cytoplasm, GO:0006355 (4) regulation of transcription, DNA-dependent, GO:0006366 (4) transcription from Pol II promoter | GO:0000074 (3) regulation of cell cycle, GO:0007165 (3) signal transduction | GO:0003700 (2) transcription factor activity, GO:0004871 (2) signal transducer activity, GO:0005515 (2) protein binding, GO:0005622 (2) intracellular, GO:0006916 (2) anti-apoptosis |
| ENSG00000121851.2 |  | GO:0003902 (6) DNA-directed RNA polymerase III activity, GO:0003900 (5) DNA-directed RNA polymerase I activity, GO:0003901 (5) DNA-directed RNA polymerase II activity, GO:0005634 (5) nucleus | GO:0006350 (4) transcription | GO:0003899 (3) DNA-directed RNA polymerase activity, GO:0005666 (3) DNA-directed RNA polymerase III complex |  |
| ENSG00000121897.2 | IPR007197 Radical SAM | GO:0005739 (6) mitochondrion | GO:0006096 (4) glycolysis | GO:0008152 (3) metabolism | GO:0006085 (2) acetyl-CoA biosynthesis, GO:0006091 (2) energy pathways, GO:0006099 (2) tricarboxylic acid cycle, GO:0006118 (2) electron transport, GO:0008415 (2) acyltransferase activity, GO:0015036 (2) disulfide oxidoreductase activity, GO:0016491 (2) oxidoreductase activity, GO:0016740 (2) transferase activity |
| ENSG00000123596.4 | IPR001202 WW/Rsp5/WWP domain, IPR000104 Antifreeze protein, type I, IPR002713 FF domain, IPR001202 WW/Rsp5/WWP domain, IPR000104 Antifreeze protein, type I, IPR002713 FF domain |  | GO:0005681 (4) spliceosome complex, GO:0006371 (4) mRNA splicing | GO:0005634 (3) nucleus, GO:0005732 (3) small nucleolar ribonucleoprotein complex, GO:0008248 (3) pre-mRNA splicing factor activity | GO:0000245 (2) spliceosome assembly |
| ENSG00000126524.1 | IPR002140 Protein of unknown function UPF0023 |  | GO:0004527 (4) exonuclease activity, GO:0006364 (4) rRNA processing | GO:0000178 (3) exosome (RNase complex), GO:0003723 (3) RNA binding, GO:0005737 (3) cytoplasm | GO:0005634 (2) nucleus, GO:0005730 (2) nucleolus |
| ENSG00000127838.3 | IPR001279 Beta-lactamase-like, IPR001279 Beta-lactamase-like | GO:0008152 (7) metabolism | GO:0005739 (4) mitochondrion | GO:0003824 (3) catalytic activity, GO:0016021 (3) integral to membrane | GO:0005215 (2) transporter activity, GO:0005634 (2) nucleus, GO:0006085 (2) acetyl-CoA biosynthesis, GO:0006810 (2) transport, GO:0008415 (2) acyltransferase activity, GO:0016740 (2) transferase activity, GO:0016787 (2) hydrolase activity |
| ENSG00000129932.1 | IPR004155 PBS lyase HEAT-like repeat, IPR000357 HEAT repeat |  |  |  | GO:0003746 (2) translation elongation factor activity, GO:0005525 (2) GTP binding, GO:0005739 (2) mitochondrion, GO:0006414 (2) translational elongation, GO:0006446 (2) regulation of translational initiation |
| ENSG00000132424.3 | IPR000694 Proline-rich region, IPR002965 Proline-rich extensin, IPR000694 Proline-rich region |  |  | GO:0005634 (3) nucleus | GO:0006355 (2) regulation of transcription, DNA-dependent, GO:0006371 (2) mRNA splicing, GO:0008248 (2) pre-mRNA splicing factor activity |
| ENSG00000132603.1 | IPR005155 Protein of unknown function UPF0113 | GO:0005634 (6) nucleus | GO:0005524 (4) ATP binding | GO:0004004 (3) ATP dependent RNA helicase activity, GO:0016787 (3) hydrolase activity | GO:0005730 (2) nucleolus, GO:0005871 (2) kinesin complex, GO:0006364 (2) rRNA processing |
| ENSG00000132768.3 | IPR002728 Diphthamide synthesis DPH2 protein |  |  | GO:0005634 (3) nucleus, GO:0016740 (3) transferase activity | GO:0003899 (2) DNA-directed RNA polymerase activity, GO:0003900 (2) DNA-directed RNA polymerase I activity, GO:0003901 (2) DNA-directed RNA polymerase II activity, GO:0003902 (2) DNA-directed RNA polymerase III activity, GO:0006350 (2) transcription, GO:0008283 (2) cell proliferation |
| ENSG00000132819.2 | IPR000504 RNA-binding region RNP-1 (RNA recognition motif) |  |  | GO:0003676 (3) nucleic acid binding | GO:0003723 (2) RNA binding, GO:0005634 (2) nucleus, GO:0006396 (2) RNA processing |
| ENSG00000133316.3 | IPR001680 G-protein beta WD-40 repeat, IPR001680 G-protein beta WD-40 repeat | GO:0005634 (7) nucleus |  |  | GO:0005524 (2) ATP binding, GO:0005871 (2) kinesin complex, GO:0006355 (2) regulation of transcription, DNA-dependent, GO:0006508 (2) proteolysis and peptidolysis |
| ENSG00000136554.1 | IPR002713 FF domain |  | GO:0005681 (4) spliceosome complex, GO:0006371 (4) mRNA splicing | GO:0005634 (3) nucleus, GO:0005732 (3) small nucleolar ribonucleoprotein complex, GO:0008248 (3) pre-mRNA splicing factor activity | GO:0000245 (2) spliceosome assembly |
| ENSG00000136718.1 | IPR007109 Brix domain, IPR001472 Bipartite nuclear localization signal | GO:0006364 (5) rRNA processing | GO:0003723 (4) RNA binding, GO:0004527 (4) exonuclease activity | GO:0000178 (3) exosome (RNase complex), GO:0005737 (3) cytoplasm | GO:0005634 (2) nucleus, GO:0005730 (2) nucleolus |
| ENSG00000136813.1 | IPR001092 Basic helix-loop-helix dimerization domain bHLH | GO:0005837 (15) 26S proteasome, GO:0005829 (13) cytosol, GO:0005634 (8) nucleus, GO:0004299 (5) proteasome endopeptidase activity, GO:0005524 (5) ATP binding, GO:0005839 (5) proteasome core complex (sensu Eukarya), GO:0006511 (5) ubiquitin-dependent protein catabolism, GO:0016787 (5) hydrolase activity |  | GO:0005838 (3) proteasome regulatory particle (sensu Eukarya), GO:0006508 (3) proteolysis and peptidolysis, GO:0008575 (3) proteasome ATPase activity | GO:0004175 (2) endopeptidase activity, GO:0005737 (2) cytoplasm |
| ENSG00000136950.1 | IPR006789 ARP2/3 complex 16 kDa subunit (p16-Arc) | GO:0005885 (6) Arp2/3 protein complex, GO:0006928 (6) cell motility |  |  | GO:0005200 (2) structural constituent of cytoskeleton, GO:0015629 (2) actin cytoskeleton |
| ENSG00000137040.1 | IPR001092 Basic helix-loop-helix dimerization domain bHLH |  |  |  | GO:0005634 (2) nucleus, GO:0005643 (2) nuclear pore, GO:0008565 (2) protein transporter activity |
| ENSG00000137054.2 |  |  | GO:0003899 (4) DNA-directed RNA polymerase activity, GO:0003900 (4) DNA-directed RNA polymerase I activity, GO:0003901 (4) DNA-directed RNA polymerase II activity, GO:0003902 (4) DNA-directed RNA polymerase III activity, GO:0005634 (4) nucleus, GO:0006350 (4) transcription |  | GO:0003677 (2) DNA binding |
| ENSG00000137942.3 | IPR001452 SH3 domain, IPR001452 SH3 domain |  |  | GO:0005739 (3) mitochondrion | GO:0006118 (2) electron transport, GO:0006886 (2) intracellular protein transport, GO:0016021 (2) integral to membrane, GO:0019866 (2) inner membrane |
| ENSG00000138442.1 | IPR001680 G-protein beta WD-40 repeat | GO:0005634 (8) nucleus, GO:0005524 (5) ATP binding | GO:0016787 (4) hydrolase activity | GO:0003676 (3) nucleic acid binding, GO:0004004 (3) ATP dependent RNA helicase activity, GO:0006412 (3) protein biosynthesis | GO:0005622 (2) intracellular, GO:0005730 (2) nucleolus, GO:0005840 (2) ribosome, GO:0005871 (2) kinesin complex, GO:0006364 (2) rRNA processing, GO:0008026 (2) ATP dependent helicase activity, GO:0016491 (2) oxidoreductase activity |
| ENSG00000143569.3 | IPR000449 Ubiquitin-associated domain, IPR001472 Bipartite nuclear localization signal |  |  |  | GO:0005634 (2) nucleus, GO:0007456 (2) eye morphogenesis (sensu Drosophila), GO:0007601 (2) vision |
| ENSG00000145332.2 | IPR006651 Kelch motif, IPR000210 BTB/POZ domain, IPR006652 Kelch repeat | GO:0004299 (6) proteasome endopeptidase activity, GO:0005829 (6) cytosol, GO:0005837 (6) 26S proteasome, GO:0005839 (6) proteasome core complex (sensu Eukarya), GO:0006511 (6) ubiquitin-dependent protein catabolism, GO:0016740 (6) transferase activity, GO:0005634 (5) nucleus, GO:0007165 (5) signal transduction |  | GO:0004713 (3) protein-tyrosine kinase activity, GO:0005524 (3) ATP binding, GO:0006468 (3) protein amino acid phosphorylation | GO:0000074 (2) regulation of cell cycle, GO:0005643 (2) nuclear pore, GO:0005737 (2) cytoplasm, GO:0006886 (2) intracellular protein transport, GO:0006950 (2) response to stress, GO:0007264 (2) small GTPase mediated signal transduction, GO:0008565 (2) protein transporter activity |
| ENSG00000146372.4 | IPR003084 Histone deacetylase, IPR000286 Histone deacetylase family, IPR003084 Histone deacetylase, IPR000286 Histone deacetylase family |  | GO:0005634 (4) nucleus | GO:0006355 (3) regulation of transcription, DNA-dependent | GO:0007049 (2) cell cycle, GO:0007275 (2) development |
| ENSG00000146963.3 | IPR004882 Protein of unknown function DUF259, IPR004882 Protein of unknown function DUF259, IPR001472 Bipartite nuclear localization signal, IPR004882 Protein of unknown function DUF259 |  | GO:0005681 (4) spliceosome complex, GO:0006371 (4) mRNA splicing | GO:0005634 (3) nucleus, GO:0005732 (3) small nucleolar ribonucleoprotein complex, GO:0008248 (3) pre-mRNA splicing factor activity | GO:0000245 (2) spliceosome assembly, GO:0003723 (2) RNA binding |
| ENSG00000147614.1 | IPR002843 H+-transporting two-sector ATPase, C (AC39) subunit | GO:0006754 (7) ATP biosynthesis, GO:0016787 (7) hydrolase activity, GO:0015992 (6) proton transport | GO:0008553 (4) hydrogen-exporting ATPase activity, phosphorylative mechanism, GO:0015078 (4) hydrogen ion transporter activity, GO:0016469 (4) proton-transporting two-sector ATPase complex | GO:0005886 (3) plasma membrane, GO:0016021 (3) integral to membrane | GO:0005215 (2) transporter activity, GO:0005224 (2) ATP-binding and phosphorylation-dependent chloride channel activity, GO:0005524 (2) ATP binding, GO:0005737 (2) cytoplasm, GO:0005753 (2) proton-transporting ATP synthase complex (sensu Eukarya), GO:0005887 (2) integral to plasma membrane, GO:0015986 (2) ATP synthesis coupled proton transport, GO:0015988 (2) energy coupled proton transport, against the electrochemical gradient |
| ENSG00000147687.4 | IPR001130 TatD-related deoxyribonuclease, IPR001130 TatD-related deoxyribonuclease |  | GO:0005524 (4) ATP binding | GO:0016740 (3) transferase activity | GO:0005739 (2) mitochondrion, GO:0006431 (2) methionyl-tRNA aminoacylation, GO:0006633 (2) fatty acid biosynthesis |
| ENSG00000148842.2 | IPR000644 CBS domain, IPR002550 CBS, IPR000644 CBS domain, IPR002550 CBS |  |  | GO:0005524 (3) ATP binding | GO:0005737 (2) cytoplasm, GO:0005739 (2) mitochondrion, GO:0006434 (2) seryl-tRNA aminoacylation, GO:0016740 (2) transferase activity, GO:0016874 (2) ligase activity |
| ENSG00000148943.3 | IPR001478 PDZ/DHR/GLGF domain, IPR004172 L27 domain |  |  | GO:0005887 (3) integral to plasma membrane, GO:0007242 (3) intracellular signaling cascade, GO:0016020 (3) membrane | GO:0005516 (2) calmodulin binding, GO:0005524 (2) ATP binding, GO:0005856 (2) cytoskeleton, GO:0006468 (2) protein amino acid phosphorylation, GO:0015629 (2) actin cytoskeleton, GO:0016740 (2) transferase activity |
| ENSG00000149532.3 | IPR000504 RNA-binding region RNP-1 (RNA recognition motif), IPR000694 Proline-rich region | GO:0005634 (5) nucleus |  | GO:0003700 (3) transcription factor activity | GO:0003676 (2) nucleic acid binding, GO:0006355 (2) regulation of transcription, DNA-dependent, GO:0006397 (2) mRNA processing, GO:0007456 (2) eye morphogenesis (sensu Drosophila), GO:0007601 (2) vision, GO:0016787 (2) hydrolase activity |
| ENSG00000153481.4 | IPR000631 Protein of unknown function UPF0031 |  |  | GO:0005524 (3) ATP binding, GO:0005634 (3) nucleus, GO:0006298 (3) mismatch repair |  |
| ENSG00000155438.1 | IPR000504 RNA-binding region RNP-1 (RNA recognition motif) | GO:0005634 (5) nucleus |  |  | GO:0005524 (2) ATP binding, GO:0005840 (2) ribosome, GO:0006412 (2) protein biosynthesis |
| ENSG00000158158.1 | IPR000644 CBS domain, IPR002550 CBS |  |  | GO:0005524 (3) ATP binding | GO:0005737 (2) cytoplasm, GO:0005739 (2) mitochondrion, GO:0006434 (2) seryl-tRNA aminoacylation, GO:0016740 (2) transferase activity, GO:0016874 (2) ligase activity |
| ENSG00000159131.3 | IPR000115 Phosphoribosylglycinamide synthetase, IPR001555 Phosphoribosylglycinamide formyltransferase, active site, IPR002376 Formyl transferase, N-terminal, IPR000728 AIR synthase related protein |  | GO:0006164 (4) purine nucleotide biosynthesis |  | GO:0006189 (2) 'de novo' IMP biosynthesis, GO:0006541 (2) glutamine metabolism, GO:0009113 (2) purine base biosynthesis, GO:0016829 (2) lyase activity |
| ENSG00000159267.4 | IPR003142 Biotin protein ligase, C-terminal, IPR004143 Biotin/lipoate A/B protein ligase domain, IPR003142 Biotin protein ligase, C-terminal, IPR004143 Biotin/lipoate A/B protein ligase domain, IPR007087 Zn-finger, C2H2 type | GO:0005739 (6) mitochondrion, GO:0005215 (5) transporter activity, GO:0005753 (5) proton-transporting ATP synthase complex (sensu Eukarya), GO:0006754 (5) ATP biosynthesis, GO:0015992 (5) proton transport, GO:0016787 (5) hydrolase activity | GO:0005624 (4) membrane fraction, GO:0015078 (4) hydrogen ion transporter activity |  | GO:0003936 (2) hydrogen-transporting two-sector ATPase activity, GO:0005224 (2) ATP-binding and phosphorylation-dependent chloride channel activity, GO:0005524 (2) ATP binding, GO:0006091 (2) energy pathways |
| ENSG00000162290.2 |  |  |  |  | GO:0000059 (2) protein-nucleus import, docking, GO:0005524 (2) ATP binding, GO:0005634 (2) nucleus, GO:0005643 (2) nuclear pore, GO:0005654 (2) nucleoplasm |
| ENSG00000162959.2 | IPR002737 Protein of unknown function DUF52 | GO:0005622 (10) intracellular, GO:0006412 (10) protein biosynthesis, GO:0003735 (5) structural constituent of ribosome, GO:0005840 (5) ribosome, GO:0016740 (5) transferase activity | GO:0003900 (4) DNA-directed RNA polymerase I activity, GO:0003901 (4) DNA-directed RNA polymerase II activity, GO:0003902 (4) DNA-directed RNA polymerase III activity, GO:0005634 (4) nucleus, GO:0005843 (4) cytosolic small ribosomal subunit (sensu Eukarya) | GO:0003899 (3) DNA-directed RNA polymerase activity, GO:0005665 (3) DNA-directed RNA polymerase II, core complex, GO:0005739 (3) mitochondrion, GO:0006350 (3) transcription, GO:0006366 (3) transcription from Pol II promoter, GO:0015935 (3) small ribosomal subunit | GO:0003677 (2) DNA binding, GO:0005777 (2) peroxisome |
| ENSG00000162961.1 | IPR007858 Dpy-30 |  |  |  | GO:0005634 (2) nucleus, GO:0008152 (2) metabolism |
| ENSG00000163481.1 | IPR001841 Zn-finger, RING |  |  |  | GO:0004842 (2) ubiquitin-protein ligase activity, GO:0006511 (2) ubiquitin-dependent protein catabolism, GO:0006512 (2) ubiquitin cycle |
| ENSG00000163745.3 | IPR001687 ATP/GTP-binding site motif A (P-loop), IPR007949 SDA1, IPR001472 Bipartite nuclear localization signal |  | GO:0005634 (4) nucleus |  | GO:0005524 (2) ATP binding, GO:0005840 (2) ribosome, GO:0006412 (2) protein biosynthesis |
| ENSG00000163938.4 | IPR001687 ATP/GTP-binding site motif A (P-loop), IPR002917 GTP-binding protein, HSR1-related, IPR001472 Bipartite nuclear localization signal, IPR001687 ATP/GTP-binding site motif A (P-loop), IPR002917 GTP-binding protein, HSR1-related, IPR001472 Bipartite nuclear localization signal | GO:0005634 (11) nucleus | GO:0003676 (4) nucleic acid binding, GO:0005524 (4) ATP binding | GO:0008026 (3) ATP dependent helicase activity | GO:0005622 (2) intracellular, GO:0005730 (2) nucleolus, GO:0005840 (2) ribosome, GO:0006355 (2) regulation of transcription, DNA-dependent, GO:0006364 (2) rRNA processing, GO:0006412 (2) protein biosynthesis, GO:0016787 (2) hydrolase activity |
| ENSG00000165271.3 | IPR005554 Nrap protein, IPR005554 Nrap protein, IPR005554 Nrap protein | GO:0005634 (8) nucleus, GO:0006364 (5) rRNA processing |  |  | GO:0005524 (2) ATP binding, GO:0005730 (2) nucleolus, GO:0005737 (2) cytoplasm, GO:0006412 (2) protein biosynthesis, GO:0007165 (2) signal transduction, GO:0016740 (2) transferase activity |
| ENSG00000166889.3 |  |  | GO:0005634 (4) nucleus, GO:0005732 (4) small nucleolar ribonucleoprotein complex, GO:0006371 (4) mRNA splicing, GO:0008248 (4) pre-mRNA splicing factor activity |  |  |
| ENSG00000167137.2 | IPR003750 Protein of unknown function DUF171 | GO:0005622 (23) intracellular, GO:0006412 (23) protein biosynthesis, GO:0005840 (17) ribosome, GO:0003735 (16) structural constituent of ribosome, GO:0005842 (9) cytosolic large ribosomal subunit (sensu Eukarya), GO:0005843 (6) cytosolic small ribosomal subunit (sensu Eukarya) |  | GO:0003723 (3) RNA binding, GO:0006413 (3) translational initiation, GO:0015934 (3) large ribosomal subunit, GO:0016020 (3) membrane | GO:0005730 (2) nucleolus, GO:0005737 (2) cytoplasm, GO:0008151 (2) cell growth and/or maintenance, GO:0016021 (2) integral to membrane |
| ENSG00000167602.1 |  | GO:0016491 (5) oxidoreductase activity | GO:0008152 (4) metabolism | GO:0005524 (3) ATP binding, GO:0005634 (3) nucleus, GO:0005739 (3) mitochondrion, GO:0005777 (3) peroxisome, GO:0006118 (3) electron transport, GO:0016787 (3) hydrolase activity | GO:0004003 (2) ATP dependent DNA helicase activity, GO:0005730 (2) nucleolus, GO:0005737 (2) cytoplasm, GO:0005759 (2) mitochondrial matrix, GO:0006091 (2) energy pathways, GO:0006289 (2) nucleotide-excision repair, GO:0006355 (2) regulation of transcription, DNA-dependent, GO:0006631 (2) fatty acid metabolism, GO:0006633 (2) fatty acid biosynthesis, GO:0008246 (2) electron transfer flavoprotein, GO:0008415 (2) acyltransferase activity, GO:0016740 (2) transferase activity |
| ENSG00000167770.2 | IPR003323 OTU-like cysteine protease |  |  |  | GO:0004842 (2) ubiquitin-protein ligase activity, GO:0006511 (2) ubiquitin-dependent protein catabolism, GO:0006512 (2) ubiquitin cycle |
| ENSG00000167797.1 |  |  |  |  | GO:0005634 (2) nucleus, GO:0006355 (2) regulation of transcription, DNA-dependent, GO:0006357 (2) regulation of transcription from Pol II promoter |
| ENSG00000167881.2 |  |  |  |  | GO:0005634 (2) nucleus, GO:0006371 (2) mRNA splicing, GO:0008248 (2) pre-mRNA splicing factor activity |
| ENSG00000167985.1 | IPR005631 Protein of unknown function DUF339 |  |  | GO:0006099 (3) tricarboxylic acid cycle, GO:0006118 (3) electron transport | GO:0005739 (2) mitochondrion, GO:0008177 (2) succinate dehydrogenase (ubiquinone) activity, GO:0009060 (2) aerobic respiration |
| ENSG00000168763.3 | IPR000644 CBS domain, IPR002550 CBS |  |  | GO:0005524 (3) ATP binding | GO:0005737 (2) cytoplasm, GO:0005739 (2) mitochondrion, GO:0006434 (2) seryl-tRNA aminoacylation, GO:0016740 (2) transferase activity, GO:0016874 (2) ligase activity |
| ENSG00000172261.1 | IPR007204 ARP2/3 complex, 21 kDa p21-Arc subunit | GO:0005885 (6) Arp2/3 protein complex, GO:0006928 (6) cell motility |  |  | GO:0005200 (2) structural constituent of cytoskeleton, GO:0015629 (2) actin cytoskeleton |
| ENSG00000175467.1 | IPR005011 SART-1 protein | GO:0006371 (5) mRNA splicing, GO:0008248 (5) pre-mRNA splicing factor activity |  | GO:0005634 (3) nucleus, GO:0005681 (3) spliceosome complex, GO:0005732 (3) small nucleolar ribonucleoprotein complex | GO:0000245 (2) spliceosome assembly |
| ENSG00000177613.2 | IPR000504 RNA-binding region RNP-1 (RNA recognition motif), IPR000504 RNA-binding region RNP-1 (RNA recognition motif) | GO:0005634 (5) nucleus |  | GO:0003723 (3) RNA binding | GO:0003676 (2) nucleic acid binding, GO:0005737 (2) cytoplasm, GO:0006378 (2) mRNA polyadenylation, GO:0006379 (2) mRNA cleavage, GO:0006397 (2) mRNA processing |
| ENSG00000178667.2 | IPR005343 Protein of unknown function UPF0120, IPR005343 Protein of unknown function UPF0120 | GO:0005634 (8) nucleus |  | GO:0004004 (3) ATP dependent RNA helicase activity, GO:0005524 (3) ATP binding | GO:0005730 (2) nucleolus, GO:0005871 (2) kinesin complex, GO:0006364 (2) rRNA processing, GO:0016787 (2) hydrolase activity |
| ENSG00000183054.1 | IPR001440 TPR repeat, IPR000156 RanBP1 domain, IPR000237 GRIP domain, IPR001440 TPR repeat |  |  |  | GO:0000074 (2) regulation of cell cycle, GO:0005634 (2) nucleus, GO:0007165 (2) signal transduction, GO:0007456 (2) eye morphogenesis (sensu Drosophila), GO:0007601 (2) vision, GO:0016740 (2) transferase activity, GO:0016787 (2) hydrolase activity |
| ENSG00000183624.1 | IPR003738 Protein of unknown function DUF159 |  |  |  | GO:0000910 (2) cytokinesis, GO:0003910 (2) DNA ligase (ATP) activity, GO:0005524 (2) ATP binding, GO:0005634 (2) nucleus, GO:0006260 (2) DNA replication, GO:0006281 (2) DNA repair, GO:0006310 (2) DNA recombination |
| ENSG00000184538.1 | IPR001440 TPR repeat |  |  |  | GO:0000074 (2) regulation of cell cycle, GO:0005634 (2) nucleus, GO:0007165 (2) signal transduction, GO:0007456 (2) eye morphogenesis (sensu Drosophila), GO:0007601 (2) vision, GO:0016740 (2) transferase activity, GO:0016787 (2) hydrolase activity |
| ENSG00000185627.2 | IPR000717 Proteasome component region PCI, IPR005820 Cation channel, non-ligand gated, IPR001682 Ca2+/Na+ channel, pore region, IPR000717 Proteasome component region PCI | GO:0005837 (14) 26S proteasome, GO:0005829 (10) cytosol, GO:0005634 (6) nucleus, GO:0005524 (5) ATP binding, GO:0016787 (5) hydrolase activity | GO:0005838 (4) proteasome regulatory particle (sensu Eukarya) | GO:0006508 (3) proteolysis and peptidolysis, GO:0008575 (3) proteasome ATPase activity | GO:0004299 (2) proteasome endopeptidase activity, GO:0005839 (2) proteasome core complex (sensu Eukarya), GO:0006511 (2) ubiquitin-dependent protein catabolism |
| ENSG00000186957.1 | IPR002494 Keratin, high sulfur B2 protein |  |  | GO:0003735 (3) structural constituent of ribosome, GO:0005524 (3) ATP binding, GO:0005622 (3) intracellular, GO:0005634 (3) nucleus, GO:0006412 (3) protein biosynthesis | GO:0005515 (2) protein binding, GO:0005529 (2) sugar binding, GO:0005737 (2) cytoplasm, GO:0005843 (2) cytosolic small ribosomal subunit (sensu Eukarya), GO:0005856 (2) cytoskeleton, GO:0005887 (2) integral to plasma membrane, GO:0006810 (2) transport, GO:0007242 (2) intracellular signaling cascade, GO:0007417 (2) central nervous system development, GO:0016020 (2) membrane, GO:0016740 (2) transferase activity, GO:0016787 (2) hydrolase activity |
| ENSG00000187239.2 | IPR001452 SH3 domain, IPR001060 Cdc15/Fes/CIP4 |  |  | GO:0005739 (3) mitochondrion | GO:0006118 (2) electron transport, GO:0006886 (2) intracellular protein transport, GO:0016021 (2) integral to membrane, GO:0019866 (2) inner membrane |
| ENSG00000187272.1 | IPR002494 Keratin, high sulfur B2 protein |  |  | GO:0003735 (3) structural constituent of ribosome, GO:0005524 (3) ATP binding, GO:0005622 (3) intracellular, GO:0005634 (3) nucleus, GO:0006412 (3) protein biosynthesis | GO:0005515 (2) protein binding, GO:0005529 (2) sugar binding, GO:0005737 (2) cytoplasm, GO:0005843 (2) cytosolic small ribosomal subunit (sensu Eukarya), GO:0005856 (2) cytoskeleton, GO:0005887 (2) integral to plasma membrane, GO:0006810 (2) transport, GO:0007242 (2) intracellular signaling cascade, GO:0007417 (2) central nervous system development, GO:0016020 (2) membrane, GO:0016740 (2) transferase activity, GO:0016787 (2) hydrolase activity |
