# Supplementary material for: A first-draft human protein-interaction map
Source: Genome Biol. 2004 Aug 13;5(9):R63. doi: 10.1186/gb-2004-5-9-r63 (PMC522870; doi:10.1186/gb-2004-5-9-r63)
Supplement: Additional data file 5 — Human disease genes with predicted protein interactions [file gb-2004-5-9-r63-s5.doc]

Supplementary table 4.

Human disease genes with predicted protein interactions. The table lists the Ensembl Ids, OMIM Ids and OMIM disease description for each gene with at least one interaction in the complete dataset. The following abbreviations from OMIM are used in the description lines: The number in parentheses after the name of each disorder indicates whether the mutation was positioned by mapping the wildtype gene (1), by mapping the disease phenotype itself (2), or by both approaches (3); {} - indicates examples of mutations that lead to universal susceptibility to a specific infection (diphtheria, polio), to frequent resistance to a specific infection (vivax malaria), protection from nicotine addiction, as well as some other susceptibilities; [] - mainly genetic variations that lead to apparently abnormal laboratory test values; ? - the equivalent of L (in limbo) for the mapping status.

| Ensembl gene | OMIM ID | Disease |
| --- | --- | --- |
| ENSG00000001084.4 | 230450 | Hemolytic anemia due to gamma-glutamylcysteine synthetase deficiency (1) |
| ENSG00000004939.3 | 109270 | [Elliptocytosis, Malaysian-Melanesian type] (3) |
| ENSG00000005271.2 | 162200 | Watson syndrome, 193520 (3) |
| ENSG00000005961.3 | 273800 | Thrombocytopenia, neonatal alloimmune (1) |
| ENSG00000006744.4 | 602759 | Prostate cancer, hereditary, 2, 176807 (2) |
| ENSG00000007168.1 | 601545 | Lissencephaly-1 (3) |
| ENSG00000007350.3 | 300044 | {?Wernicke-Korsakoff syndrome, susceptibility to} (1) |
| ENSG00000007372.4 | 106210 | Peters anomaly (3) |
| ENSG00000007933.1 | 136132 | [Fish-odor syndrome], 602079 (3) |
| ENSG00000009709.1 | 167410 | Rhabdomyosarcoma, alveolar, 268220 (3) |
| ENSG00000010671.3 | 300300 | XLA and isolated growth hormone deficiency, 307200 (3) (?) |
| ENSG00000011052.2 | 156490 | Neuroblastoma (3) |
| ENSG00000012048.3 | 113705 | Ovarian cancer (3) |
| ENSG00000012223.2 | 150210 | Lactoferrin-deficient neutrophils, 245480 (1) (?) |
| ENSG00000015285.2 | 301000 | Wiskott-Aldrich syndrome (3) |
| ENSG00000018280.2 | 600266 | Resistance/susceptibility to TB, etc. (1) (?) |
| ENSG00000021488.2 | 600918 | Cystinuria, type III (2) |
| ENSG00000021574.2 | 182601 | Spastic paraplegia-4 (3) |
| ENSG00000021852.1 | 120960 | C8 deficiency, type II (3) |
| ENSG00000023228.2 | 157655 | Lactic acidosis due to defect in iron-sulfur cluster of complex I (1) |
| ENSG00000032514.1 | 133540 | Cockayne syndrome-2, late onset (2) |
| ENSG00000036473.1 | 311250 | Ornithine transcarbamylase deficiency (3) |
| ENSG00000039068.3 | 192090 | Ovarian carcinoma (3) |
| ENSG00000039537.2 | 217050 | Combined C6/C7 deficiency (1) |
| ENSG00000044446.2 | 306000 | Glycogenosis, X-linked hepatic, type II (3) |
| ENSG00000047249.3 | 188826 | Sorsby fundus dystrophy, 136900 (3) |
| ENSG00000047343.3 | 160777 | Griscelli disease, 214450 (3) |
| ENSG00000049319.1 | 264600 | Pseudovaginal perineoscrotal hypospadias (3) |
| ENSG00000049496.1 | 238300 | Hyperglycinemia, nonketotic, type I (3) |
| ENSG00000054598.1 | 601090 | Iridogoniodysgenesis, 601631 (3) |
| ENSG00000056345.1 | 173470 | Glanzmann thrombasthenia, type B (3) |
| ENSG00000058272.3 | 157900 | Moebius syndrome (2) (?) |
| ENSG00000059377.4 | 274180 | Thromboxane synthase deficiency (2) |
| ENSG00000059573.1 | 138250 | P5CS deficiency (1) (?) |
| ENSG00000060982.3 | 113520 | Hyperleucinemia-isoleucinemia or hypervalinemia (1) (?) |
| ENSG00000063854.1 | 138760 | [Glyoxalase II deficiency] (1) |
| ENSG00000064195.1 | 600525 | Trichodontoosseous syndrome, 190320 (3) |
| ENSG00000064601.2 | 256540 | Galactosialidosis (3) |
| ENSG00000065154.1 | 258870 | Gyrate atrophy of choroid and retina with ornithinemia, B6 responsive or unresponsive (3) |
| ENSG00000065883.2 | 121700 | Congenital hereditary endothelial dystrophy of cornea (2) |
| ENSG00000066926.1 | 177000 | Protoporphyria, erythropoietic, recessive, with liver failure (3) |
| ENSG00000067082.3 | 601188 | Prostate adenocarcinoma (2) |
| ENSG00000067177.2 | 311870 | Muscle glycogenosis (3) |
| ENSG00000067715.3 | 600192 | Sarcoma, synovial (1) |
| ENSG00000067955.4 | 121360 | Myeloid leukemia, acute, M4Eo subtype (2) |
| ENSG00000068976.2 | 232600 | McArdle disease (3) |
| ENSG00000069399.1 | 109560 | Leukemia/lymphoma, B-cell, 3 (2) |
| ENSG00000069696.3 | 126452 | [Novelty seeking personality] (1) |
| ENSG00000071537.2 | 601458 | Inflammatory bowel disease-2 (2) |
| ENSG00000071564.2 | 147141 | Leukemia, acute lymphoblastic (1) |
| ENSG00000072210.3 | 270200 | Sjogren-Larsson syndrome (3) |
| ENSG00000072778.3 | 201475 | VLCAD deficiency (3) |
| ENSG00000073578.2 | 600857 | Leigh syndrome (3) |
| ENSG00000074319.1 | 601387 | Breast cancer (3) |
| ENSG00000074695.1 | 601567 | Combined factor V and VIII deficiency, 227300 (3) |
| ENSG00000074800.3 | 172430 | Enolase deficiency (1) |
| ENSG00000075043.4 | 118504 | Epilepsy, nocturnal frontal lobe, 600513 (3) |
| ENSG00000075239.2 | 203750 | 3-ketothiolase deficiency (3) |
| ENSG00000076242.1 | 120436 | Turcot syndrome with glioblastoma, 276300 (3) |
| ENSG00000077279.4 | 300067 | Subcortical laminar heterotopia, X-linked dominant (2) |
| ENSG00000077498.2 | 203100 | Waardenburg syndrome/ocular albinism, digenic, 103470 (3) |
| ENSG00000078900.1 | 601990 | Neuroblastoma (1) (?) |
| ENSG00000079482.2 | 300127 | Mental retardation, X-linked, 60 (3) |
| ENSG00000080618.2 | 212070 | Carboxypeptidase B deficiency (1) |
| ENSG00000080815.2 | 227646 | Fanconi anemia, type D (2) |
| ENSG00000080819.1 | 303400 | Cleft palate, X-linked (2) |
| ENSG00000083123.2 | 248611 | Maple syrup urine disease, type Ib (3) |
| ENSG00000083720.1 | 245050 | Ketoacidosis due to SCOT deficiency (3) |
| ENSG00000083799.4 | 132700 | Cylindromatosis (2) |
| ENSG00000084110.2 | 235800 | [Histidinemia] (1) |
| ENSG00000084674.2 | 107730 | Hypobetalipoproteinemia (3) |
| ENSG00000084754.1 | 600890 | Mitochondrial trifunctional protein deficiency (1) |
| ENSG00000084764.1 | 312610 | Retinitis pigmentosa-3 (3) |
| ENSG00000085117.1 | 600623 | Prostate cancer, 176807 (2) |
| ENSG00000085276.3 | 165215 | 3q21q26 syndrome (1) |
| ENSG00000085662.2 | 313700 | Spinal and bulbar muscular atrophy of Kennedy, 313200 (3) |
| ENSG00000087053.2 | 601382 | Charcot-Marie-Tooth neuropathy-4B (2) |
| ENSG00000087086.1 | 134790 | Hyperferritinemia-cataract syndrome, 600886 (3) |
| ENSG00000087460.4 | 139320 | Somatotrophinoma (3) |
| ENSG00000089232.2 | 601517 | Spinocerebellar ataxia-2, 183090 (3) |
| ENSG00000090382.1 | 153450 | Amyloidosis, renal, 105200 (3) |
| ENSG00000091136.2 | 150240 | Cutis laxa, marfanoid neonatal type (1) (?) |
| ENSG00000091137.1 | 274600 | Pendred syndrome (3) |
| ENSG00000091138.1 | 126650 | Colon cancer (1) (?) |
| ENSG00000091140.1 | 600065 | Leukocyte adhesion deficiency, 116920 (3) |
| ENSG00000091409.3 | 147556 | Epidermolysis bullosa, junctional, with pyloric stenosis, 226730 (3) |
| ENSG00000091483.1 | 136850 | Fumarase deficiency (3) |
| ENSG00000091513.2 | 190000 | Atransferrinemia (1) |
| ENSG00000091536.3 | 602666 | Deafness, autosomal recessive 3, 600316 (3) |
| ENSG00000092054.2 | 160760 | Central core disease, one form (3) (?) |
| ENSG00000092295.1 | 190195 | Ichthyosis, lamellar, autosomal recessive, 242300 (3) |
| ENSG00000092529.4 | 114240 | Muscular dystrophy, limb-girdle, type 2A, 253600 (3) |
| ENSG00000095002.1 | 120435 | Muir-Torre syndrome, 158320 (3) |
| ENSG00000095321.2 | 600184 | Carnitine acetyltransferase deficiency (1) (?) |
| ENSG00000096006.1 | 600593 | Craniosynostosis, Adelaide type (2) |
| ENSG00000097007.1 | 189980 | Leukemia, chronic myeloid (3) |
| ENSG00000100031.4 | 231950 | Glutathioninuria (1) |
| ENSG00000100033.3 | 239500 | Hyperprolinemia, type I (1) |
| ENSG00000100177.2 | 104170 | Schindler disease (3) |
| ENSG00000100197.4 | 124030 | {?Parkinsonism, susceptibility to} (1) |
| ENSG00000100243.4 | 250800 | Methemoglobinemia, type II (3) |
| ENSG00000100357.2 | 103050 | Autism, succinylpurinemic (3) |
| ENSG00000100504.2 | 232700 | Glycogen storage disease VI (3) |
| ENSG00000100831.1 | 164050 | Nucleoside phosphorylase deficiency, immunodeficiency due to (3) |
| ENSG00000100836.1 | 602279 | Oculopharyngeal muscular dystrophy, autosomal recessive, 257950 (3) |
| ENSG00000101076.3 | 600281 | Non-insulin-dependent diabetes mellitus, 125853 (3) |
| ENSG00000101093.2 | 102700 | Severe combined immunodeficiency due to ADA deficiency (3) |
| ENSG00000101204.1 | 602235 | Epilepsy, benign, neonatal, type 1, 121200 (3) |
| ENSG00000101246.3 | 601916 | Pancreatic cancer (2) |
| ENSG00000101384.1 | 601920 | Alagille syndrome, 118450 (3) |
| ENSG00000101868.2 | 312040 | N syndrome, 310465 (1) (?) |
| ENSG00000101986.2 | 300100 | Adrenomyeloneuropathy (3) |
| ENSG00000102022.2 | 308840 | Spastic paraplegia, 312900 (3) |
| ENSG00000102081.2 | 309550 | Fragile X syndrome (3) |
| ENSG00000102125.4 | 302060 | Noncompaction of left ventricular myocardium, isolated (3) |
| ENSG00000102144.2 | 311800 | Myoglobinuria/hemolysis due to PGK deficiency (3) |
| ENSG00000102314.2 | 312760 | Turner syndrome (1) |
| ENSG00000102683.1 | 253700 | Muscular dystrophy, limb-girdle, type 2C (3) |
| ENSG00000103051.2 | 304020 | Cone dystrophy, progressive X-linked, 1 (2) |
| ENSG00000103080.1 | 245900 | Norum disease (3) |
| ENSG00000103449.1 | 602218 | Townes-Brocks syndrome, 107480 (3) |
| ENSG00000103876.1 | 276700 | Tyrosinemia, type I (3) |
| ENSG00000104044.3 | 203200 | Albinism, oculocutaneous, type II (3) |
| ENSG00000104313.2 | 601653 | Branchiootorenal syndrome, 113650 (3) |
| ENSG00000104687.2 | 138300 | Hemolytic anemia due to glutathione reductase deficiency (1) |
| ENSG00000104774.1 | 248500 | Mannosidosis, alpha- (3) |
| ENSG00000104812.2 | 138570 | {Non-insulin dependent diabetes mellitus, susceptibility to} (2) |
| ENSG00000104884.2 | 126340 | Xeroderma pigmentosum, group D, 278730 (3) |
| ENSG00000104903.2 | 151440 | Leukemia, T-cell acute lymphoblastoid (2) |
| ENSG00000105220.2 | 172400 | Hydrops fetalis, one form (1) |
| ENSG00000105221.2 | 164731 | Ovarian carcinoma, 167000 (2) |
| ENSG00000105379.1 | 130410 | Glutaricaciduria, type IIB (3) |
| ENSG00000105392.1 | 602225 | Leber congenital amaurosis, type III (3) |
| ENSG00000105486.3 | 126391 | DNA ligase I deficiency (3) |
| ENSG00000105552.4 | 113530 | Hypervalinemia or hyperleucine-isoleucinemia (1) (?) |
| ENSG00000105607.3 | 231670 | Glutaricaciduria, type I (3) |
| ENSG00000105618.3 | 600138 | Retinitis pigmentosa-11 (2) |
| ENSG00000105641.1 | 601843 | Hypothyroidism, congenital, 274400 (3) |
| ENSG00000105664.1 | 600310 | Pseudoachondroplasia, 177170 (3) |
| ENSG00000105953.3 | 203740 | Alpha-ketoglutarate dehydrogenase deficiency (1) |
| ENSG00000106125.4 | 107776 | Colton blood group, 110450 (3) |
| ENSG00000106571.1 | 165240 | Postaxial polydactyly type A1, 174200 (3) |
| ENSG00000106588.1 | 147020 | Agammaglobulinemia, 601495 (3) |
| ENSG00000106633.3 | 138079 | MODY, type 2, 125851 (3) |
| ENSG00000106782.2 | 200150 | Choreoacanthocytosis (2) |
| ENSG00000107165.1 | 115501 | Albinism, rufous, 278400 (3) |
| ENSG00000107798.4 | 278000 | Wolman disease (3) |
| ENSG00000107807.1 | 186770 | Leukemia, T-cell acute lymphocytic (2) |
| ENSG00000108784.1 | 252920 | Sanfilippo syndrome, type B (3) |
| ENSG00000109906.3 | 176797 | Leukemia, acute promyelocytic, PL2F/RARA type (3) |
| ENSG00000110090.1 | 600528 | CPT deficiency, hepatic, type I, 255120 (1) |
| ENSG00000110911.4 | 600795 | Dementia, familial, nonspecific (2) |
| ENSG00000110921.2 | 251170 | Mevalonicaciduria (3) |
| ENSG00000111012.1 | 264700 | Pseudo-vitamin D dependency rickets 1 (2) |
| ENSG00000111262.1 | 176260 | Episodic ataxia/myokymia syndrome, 160120 (3) |
| ENSG00000111275.3 | 100650 | {?Fetal alcohol syndrome} (1) |
| ENSG00000111319.2 | 600228 | Pseudohypoaldosteronism, type I, 264350 (3) |
| ENSG00000111664.1 | 139130 | {Hypertension, essential, susceptibility to}, 145500 (3) |
| ENSG00000111669.1 | 190450 | Hemolytic anemia due to triosephosphate isomerase deficiency (3) |
| ENSG00000111676.3 | 125370 | Dentatorubro-pallidoluysian atrophy (3) |
| ENSG00000111713.1 | 138571 | Glycogen synthase, liver, deficiency of, 240600 (1) |
| ENSG00000111716.1 | 150100 | Lactate dehydrogenase-B deficiency (3) |
| ENSG00000112041.1 | 602280 | Retinitis pigmentosa-14, 600132 (3) |
| ENSG00000112077.2 | 180297 | Anemia, hemolytic, Rh-null, suppressor type, 268150 (3) |
| ENSG00000112319.4 | 601316 | Deafness, autosomal dominant 10 (2) |
| ENSG00000112357.1 | 601757 | Rhizomelic chondrodysplasia punctata, type 1, 215100 (3) |
| ENSG00000112936.4 | 217070 | C7 deficiency (1) |
| ENSG00000113318.2 | 600887 | Endometrial carcinoma (3) |
| ENSG00000113552.4 | 172400 | Hydrops fetalis, one form (1) |
| ENSG00000113600.1 | 120940 | C9 deficiency (3) |
| ENSG00000113889.1 | 228960 | [Kininogen deficiency] (3) |
| ENSG00000113916.3 | 109565 | Lymphoma, diffuse large cell (3) |
| ENSG00000113924.3 | 203500 | Alkaptonuria (3) |
| ENSG00000114054.2 | 232050 | Propionicacidemia, type II or pccB type (3) |
| ENSG00000114125.1 | 181031 | Oguchi disease-1, 258100 (3) |
| ENSG00000114349.1 | 139330 | Night blindness, congenital stationary (3) |
| ENSG00000114353.3 | 139360 | Pituitary ACTH-secreting adenoma (3) |
| ENSG00000114480.1 | 232500 | Glycogen storage disease IV (3) |
| ENSG00000114491.2 | 258900 | Oroticaciduria (3) |
| ENSG00000115159.2 | 138430 | Diabetes mellitus, type II (3) |
| ENSG00000115844.1 | 600202 | Dyslexia, specific, 2 (2) |
| ENSG00000115904.1 | 135300 | Fibromatosis, gingival (2) |
| ENSG00000116062.1 | 600678 | {Cancer susceptibility} (3) |
| ENSG00000116703.3 | 118800 | Choreoathetosis, familial paroxysmal (2) |
| ENSG00000116745.3 | 180069 | Retinitis pigmentosa-20 (3) |
| ENSG00000116761.1 | 219500 | [Cystathioninuria] (1) |
| ENSG00000116984.1 | 156570 | Methylcobalamin deficiency, cbl G type (3) |
| ENSG00000117013.2 | 600101 | Deafness, autosomal dominant 2 (2) |
| ENSG00000117054.1 | 201450 | Acyl-CoA dehydrogenase, medium chain, deficiency of (3) |
| ENSG00000117118.1 | 185470 | Myopathy due to succinate dehydrogenase deficiency (1) (?) |
| ENSG00000117308.2 | 230350 | Galactose epimerase deficiency (3) |
| ENSG00000117394.3 | 138140 | Glucose transport defect, blood-brain barrier (3) |
| ENSG00000117528.1 | 170995 | Zellweger syndrome-2 (3) |
| ENSG00000117601.2 | 107300 | Antithrombin III deficiency (3) |
| ENSG00000118194.4 | 191045 | Cardiomyopathy, familial hypertrophic, 2, 115195 (3) |
| ENSG00000118271.1 | 176300 | [Dystransthyretinemic hyperthyroxinemia](3) |
| ENSG00000118402.1 | 600110 | Stargardt disease-3 (2) |
| ENSG00000118520.2 | 207800 | Argininemia (3) |
| ENSG00000119048.1 | 179095 | Male infertility (1) (?) |
| ENSG00000119508.4 | 600542 | Chondrosarcoma, extraskeletal myxoid (1) |
| ENSG00000119537.2 | 136440 | Lymphoma/leukemia, B-cell, variant (1) |
| ENSG00000119950.4 | 600020 | Prostate cancer, 176807 (3) |
| ENSG00000120149.1 | 168500 | Parietal foramina (2) |
| ENSG00000120437.2 | 100678 | ACAT2 deficiency (1) (?) |
| ENSG00000121691.1 | 115500 | Acatalasemia (3) |
| ENSG00000121741.3 | 602221 | Stem-cell leukemia/lymphoma syndrome (3) |
| ENSG00000122126.3 | 309000 | Lowe syndrome (3) |
| ENSG00000122512.3 | 600259 | Turcot syndrome with glioblastoma, 276300 (3) |
| ENSG00000122877.1 | 129010 | Neuropathy, congenital hypomyelinating, 1 (3) |
| ENSG00000123191.2 | 277900 | Wilson disease (3) |
| ENSG00000123983.2 | 601622 | Saethre-Chotzen syndrome, 101400 (3) |
| ENSG00000124253.2 | 261680 | Hypoglycemia due to PCK1 deficiency (1) (?) |
| ENSG00000124299.2 | 170100 | Prolidase deficiency (3) |
| ENSG00000124491.1 | 134570 | Factor XIIIA deficiency (3) |
| ENSG00000124587.3 | 601498 | Peroxisomal biogenesis disorder, complementation group 4 (3) |
| ENSG00000124659.1 | 163950 | Noonan syndrome-1 (2) |
| ENSG00000124795.2 | 125264 | Leukemia, acute nonlymphocytic (2) |
| ENSG00000124812.4 | 601916 | Pancreatic cancer (2) |
| ENSG00000124813.3 | 600211 | Cleidocranial dysplasia, 119600 (3) |
| ENSG00000125378.2 | 112262 | Fibrodysplasia ossificans progressiva, 135100 (1) (?) |
| ENSG00000125482.2 | 600635 | Goiter, familial, due to TTF-1 defect (1) |
| ENSG00000125779.4 | 234200 | Neurodegeneration with brain iron accumulation (2) |
| ENSG00000125814.4 | 162100 | Neuralgic amyotrophy with predilection for brachial plexus (2) |
| ENSG00000125845.1 | 112261 | Fibrodysplasia ossificans progressiva (1) (?) |
| ENSG00000126088.1 | 176100 | Porphyria, hepatoerythropoietic (3) |
| ENSG00000126883.2 | 114350 | Leukemia, acute myeloid (2) |
| ENSG00000127980.2 | 602136 | Zellweger syndrome-1, 214100 (3) |
| ENSG00000128309.2 | 159595 | Leukemia, transient, of Down syndrome (2) |
| ENSG00000128311.2 | 179605 | Retinitis punctata albescens (3) |
| ENSG00000128573.4 | 602081 | Speech-language disorder-1 (2) |
| ENSG00000128683.1 | 266100 | Pyridoxine dependency with seizures (1) (?) |
| ENSG00000128739.4 | 182279 | Prader-Willi syndrome (1) (?) |
| ENSG00000129084.3 | 142360 | Thrombophilia due to heparin cofactor II deficiency (3) |
| ENSG00000129562.1 | 600243 | Temperature-sensitive apoptosis (1) |
| ENSG00000129991.1 | 191044 | Cardiomyopathy, familial hypertrophic (3) |
| ENSG00000130164.1 | 143890 | Hypercholesterolemia, familial (3) |
| ENSG00000130654.2 | 141800 | Thalassemias, alpha- (3) |
| ENSG00000130707.2 | 215700 | Citrullinemia (3) |
| ENSG00000130826.2 | 300126 | Dyskeratosis congenita-1, 305000 (3) |
| ENSG00000130948.1 | 264300 | Pseudohermaphroditism, male, with gynecomastia (3) |
| ENSG00000131238.1 | 600722 | Ceroid lipofuscinosis, neuronal-1, infantile, 256730 (3) |
| ENSG00000131504.2 | 602121 | Deafness, autosomal dominant nonsyndromic sensorineural, 1, 124900 (3) |
| ENSG00000131828.2 | 312170 | Pyruvate dehydrogenase deficiency (3) |
| ENSG00000131979.2 | 600225 | Phenylketonuria, atypical, due to GCH1 deficiency, 233910 (1) |
| ENSG00000132142.4 | 200350 | Acetyl-CoA carboxylase deficiency (1) |
| ENSG00000132438.4 | 310200 | Duchenne muscular dystrophy (3) |
| ENSG00000133103.3 | 300085 | Cone dystrophy, progressive X-linked, 2 (2) |
| ENSG00000133112.3 | 190605 | Triphalangeal thumb-polysyndactyly syndrome (2) |
| ENSG00000133703.1 | 190070 | Colorectal cancer (1) |
| ENSG00000133835.1 | 601860 | D-bifunctional protein deficiency (3) |
| ENSG00000134240.1 | 600234 | HMG-CoA synthease-2 deficiency (1) |
| ENSG00000134242.1 | 308240 | Lymphoproliferative syndrome, X-linked (2) |
| ENSG00000134333.1 | 150000 | Exertional myoglobinuria due to deficiency of LDH-A (3) |
| ENSG00000134899.1 | 133530 | Xeroderma pigmentosum, group G, 278780 (3) |
| ENSG00000134982.2 | 175100 | Turcot syndrome, 276300 (3) |
| ENSG00000135069.2 | 181030 | Salivary gland pleomorphic adenoma (2) |
| ENSG00000135111.2 | 601621 | Ulnar-mammary syndrome, 181450 (3) |
| ENSG00000135424.3 | 600536 | Myopathy, congenital (3) |
| ENSG00000135446.4 | 123829 | Melanoma (3) |
| ENSG00000135605.1 | 148500 | Tylosis with esophageal cancer (2) |
| ENSG00000135903.3 | 193500 | Waardenburg syndrome, type III, 148820 (3) |
| ENSG00000135929.1 | 213700 | Cerebrotendinous xanthomatosis (3) |
| ENSG00000136523.3 | 165500 | Optic atrophy 1 (2) |
| ENSG00000136872.4 | 229600 | Fructose intolerance (3) |
| ENSG00000136936.1 | 278700 | Xeroderma pigmentosum, group A (3) |
| ENSG00000136944.1 | 602575 | Nail-patella syndrome, 161200 (3) |
| ENSG00000136997.1 | 190080 | Burkitt lymphoma (3) |
| ENSG00000137104.2 | 230400 | Galactosemia (3) |
| ENSG00000137474.2 | 276903 | Usher syndrome, type 1B (3) |
| ENSG00000137992.1 | 248610 | Maple syrup urine disease, type II (3) |
| ENSG00000138029.2 | 143450 | Trifunctional protein deficiency, type II (3) |
| ENSG00000138030.2 | 229800 | [Fructosuria] (1) |
| ENSG00000138079.1 | 104614 | Cystinuria, 220100 (3) |
| ENSG00000138829.1 | 121050 | Contractural arachnodactyly, congenital (3) |
| ENSG00000139083.2 | 600618 | Leukemia, acute lymphoblastic (1) |
| ENSG00000139197.1 | 600414 | Adrenoleukodystrophy, neonatal, 202370 (3) |
| ENSG00000139567.1 | 601284 | Hereditary hemorrhagic telangiectasia-2, 600376 (3) |
| ENSG00000140263.1 | 182500 | Cataract, congenital (2) (?) |
| ENSG00000140374.3 | 231680 | Glutaricaciduria, type IIA (1) |
| ENSG00000140416.4 | 191010 | Cardiomyopathy, familial hypertrophic, 3, 115196 (3) |
| ENSG00000140650.2 | 601785 | Carbohydrate-deficient glycoprotein syndrome, type I, 212065 (3) |
| ENSG00000140824.2 | 276600 | Tyrosinemia, type II (3) |
| ENSG00000141018.3 | 602783 | Spastic paraplegia-7 (3) |
| ENSG00000141037.3 | 155555 | {UV-induced skin damage, vulnerability to} (3) |
| ENSG00000141279.2 | 181030 | Salivary gland pleomorphic adenoma (2) |
| ENSG00000141380.4 | 600192 | Sarcoma, synovial (1) |
| ENSG00000141510.1 | 191170 | Li-Fraumeni syndrome (3) |
| ENSG00000141646.1 | 600993 | Pancreatic cancer (3) |
| ENSG00000141736.1 | 256550 | Sialidosis, type II (3) |
| ENSG00000141744.1 | 171190 | Hypertension, essential, 145500 (1) (?) |
| ENSG00000141959.3 | 171860 | Hemolytic anemia due to phosphofructokinase deficiency (1) |
| ENSG00000141985.1 | 601768 | Leukemia, acute myeloid (3) |
| ENSG00000142046.2 | 248600 | Maple syrup urine disease, type Ia (3) |
| ENSG00000142168.2 | 147450 | Amytrophic lateral sclerosis, due to SOD1 deficiency, 105400 (3) |
| ENSG00000142173.3 | 120240 | Bethlem myopathy, 158810 (3) |
| ENSG00000142192.2 | 104760 | Schizophrenia, chronic (3) |
| ENSG00000142599.4 | 601916 | Pancreatic cancer (2) |
| ENSG00000142910.2 | 207800 | Argininemia (3) |
| ENSG00000142954.3 | 274270 | {Fluorouracil toxicity, sensitivity to} (1) |
| ENSG00000143224.2 | 600923 | Porphyria variegata, 176200 (3) |
| ENSG00000143549.4 | 191030 | Nemaline myopathy-1, 161800 (3) |
| ENSG00000143627.3 | 266200 | Anemia, hemolytic, due to PK deficiency (3) |
| ENSG00000143669.1 | 214500 | Chediak-Higashi syndrome (3) |
| ENSG00000143801.2 | 600759 | Alzheimer disease-4 (3) |
| ENSG00000143819.1 | 132810 | Fetal hydantoin syndrome (1) (?) |
| ENSG00000143839.4 | 179820 | [Hyperproreninemia] (3) |
| ENSG00000144212.4 | 104311 | Alzheimer disease-3 (3) |
| ENSG00000144231.1 | 180250 | Retinol binding protein, deficiency of (1) (?) |
| ENSG00000144580.2 | 180020 | Retinal cone dystrophy-1 (2) (?) |
| ENSG00000145020.2 | 238310 | Hyperglycinemia, nonketotic, type II (1) |
| ENSG00000145050.1 | 601916 | Pancreatic cancer (2) |
| ENSG00000145362.1 | 600919 | Long QT syndrome-4 with sinus bradycardia (2) |
| ENSG00000145888.3 | 138491 | Startle disease/hyperekplexia, autosomal dominant, 149400 (3) |
| ENSG00000146085.1 | 251000 | Methylmalonicaciduria, mutase deficiency type (3) |
| ENSG00000147224.1 | 311850 | Phosphoribosyl pyrophosphate synthetase-related gout (3) |
| ENSG00000147257.2 | 300037 | Simpson dysmorphia syndrome, 312870 (3) |
| ENSG00000148218.1 | 125270 | {Lead poisoning, susceptibility to} (3) |
| ENSG00000148400.1 | 190198 | Leukemia, T-cell acute lymphoblastic (2) |
| ENSG00000148672.1 | 138130 | Hyperinsulinism-hyperammonemia syndrome (3) |
| ENSG00000148732.2 | 176801 | Metachromatic leukodystrophy due to deficiency of SAP-1 (3) |
| ENSG00000149397.2 | 176000 | Porphyria, acute intermittent (3) |
| ENSG00000149925.3 | 103850 | Aldolase A deficiency (3) |
| ENSG00000150275.3 | 602083 | Usher syndrome, type IF (2) |
| ENSG00000150781.1 | 168000 | Paraganglioma, familial nonchromaffin, 1 (2) |
| ENSG00000150787.1 | 261640 | Phenylketonuria due to PTS deficiency (3) |
| ENSG00000150907.1 | 136533 | Rhabdomyosarcoma, alveolar, 268220 (3) |
| ENSG00000151224.1 | 250850 | Hypermethioninemia, persistent, autosomal dominant, due to methionine adenosyltransferase I/III deficiency (3) |
| ENSG00000151552.1 | 261630 | Phenylketonuria due to dihydropteridine reductase deficiency (3) |
| ENSG00000151729.1 | 601226 | Progressive external ophthalmoplegia, type 2 (2) |
| ENSG00000151747.2 | 182600 | Spastic paraplegia-3A (2) |
| ENSG00000151849.2 | 150270 | Laryngeal adductor paralysis (2) (?) |
| ENSG00000152433.4 | 313400 | Spondyloepiphyseal dysplasia tarda (2) |
| ENSG00000152556.3 | 232800 | Glycogen storage disease VII (3) |
| ENSG00000152591.2 | 125490 | Dentinogenesis imperfecta-1 (2) |
| ENSG00000154229.2 | 176960 | Pituitary tumor, invasive (3) |
| ENSG00000154646.2 | 226200 | Enterokinase deficiency (1) |
| ENSG00000154767.1 | 278720 | Xeroderma pigmentosum, group C (3) |
| ENSG00000155465.1 | 222700 | Lysinuric protein intolerance (2) |
| ENSG00000155966.2 | 309548 | Mental retardation, X-linked, FRAXE type (3) |
| ENSG00000156052.1 | 600998 | Bleeding diathesis due to GNAQ deficiency (1) |
| ENSG00000156515.4 | 142600 | Hemolytic anemia due to hexokinase deficiency (3) |
| ENSG00000156873.4 | 172471 | Glycogenosis, hepatic, autosomal (3) |
| ENSG00000156925.2 | 306955 | Heterotaxy, X-linked visceral (3) |
| ENSG00000157131.3 | 120950 | C8 deficiency, type I (2) |
| ENSG00000157168.3 | 142640 | Thrombophilia due to elevated HRG (1) (?) |
| ENSG00000157184.1 | 600650 | Myopathy due to CPT II deficiency, 255110 (3) |
| ENSG00000157562.4 | 303630 | Leiomyomatosis-nephropathy syndrome, 308940 (1) |
| ENSG00000158104.3 | 122000 | Corneal dystrophy, posterior polymorphous (2) |
| ENSG00000158125.2 | 278300 | Xanthinuria, type I (3) |
| ENSG00000158578.4 | 301300 | Anemia, sideroblastic/hypochromic (3) |
| ENSG00000158581.3 | 102600 | Urolithiasis, 2,8-dihydroxyadenine (3) |
| ENSG00000159023.3 | 130500 | Elliptocytosis-1 (3) |
| ENSG00000159216.4 | 151385 | Leukemia, acute myeloid (3) |
| ENSG00000159251.1 | 102540 | Cardiomyopathy, idiopathic dilated (3) |
| ENSG00000159267.4 | 253270 | Multiple carboxylase deficiency, biotin-responsive (3) |
| ENSG00000159640.2 | 106180 | {Myocardial infarction, susceptibility to} (3) |
| ENSG00000160200.4 | 236200 | Homocystinuria, B6-responsive and nonresponsive types (3) |
| ENSG00000160202.1 | 123580 | Cataract, congenital, autosomal dominant (3) |
| ENSG00000160211.1 | 305900 | Hemolytic anemia due to G6PD deficiency (3) |
| ENSG00000160255.4 | 600065 | Leukocyte adhesion deficiency, 116920 (3) |
| ENSG00000160808.1 | 160790 | Cardiomopathy, hypertrophic, mid-ventricular chamber type (3) |
| ENSG00000161280.3 | 141900 | Thalassemias, beta- (3) |
| ENSG00000162367.1 | 187040 | Leukemia-1, T-cell acute lymphoblastic (3) |
| ENSG00000162374.3 | 168360 | Paraneoplastic sensory neuropathy (1) |
| ENSG00000162688.3 | 232400 | Glycogen storage disease IIIb (3) |
| ENSG00000163161.1 | 133510 | Xeroderma pigmentosum, group B (3) |
| ENSG00000163581.2 | 138160 | Fanconi-Bickel syndrome, 227810 (3) |
| ENSG00000164025.4 | 103720 | {Alcoholism, susceptibility to} (1) |
| ENSG00000164258.1 | 602694 | Complex I deficiency (3) |
| ENSG00000164708.1 | 261670 | Myopathy due to phosphoglycerate mutase deficiency (3) |
| ENSG00000164741.3 | 140100 | [Hypohaptogloginemia] (3) |
| ENSG00000164751.4 | 170993 | Zellweger syndrome-3 (3) |
| ENSG00000165060.1 | 227650 | Fanconi anemia, type A (3) |
| ENSG00000165140.1 | 229700 | Fructose-bisphosphatase deficiency (1) |
| ENSG00000165168.1 | 306400 | Chronic granulomatous disease, X-linked (3) |
| ENSG00000165240.2 | 300011 | Occipital horn syndrome, 304150 (3) |
| ENSG00000165699.1 | 600968 | Gitelman syndrome, 263800 (3) |
| ENSG00000165704.2 | 308000 | Lesch-Nyhan syndrome (3) |
| ENSG00000166147.2 | 134797 | Shprintzen-Goldberg syndrome, 182212 (3) |
| ENSG00000166311.1 | 257200 | Niemann-Pick disease, type B (3) |
| ENSG00000166347.2 | 250790 | Methemoglobinemia due to cytochrome b5 deficiency (3) |
| ENSG00000166828.1 | 600761 | Pseudohypoaldosteronism, type I, 264350 (3) |
| ENSG00000166974.2 | 180100 | Retinitis pigmentosa-1 (2) |
| ENSG00000167085.1 | 176705 | Breast cancer, sporadic (3) |
| ENSG00000167165.3 | 191740 | [Gilbert syndrome], 143500 (3) |
| ENSG00000167580.1 | 107777 | Diabetes insipidus, nephrogenic, autosomal recessive, 222000 (3) |
| ENSG00000167780.1 | 100678 | ACAT2 deficiency (1) (?) |
| ENSG00000167986.1 | 600045 | Xeroderma pigmentosum, group E, subtype 2 (1) |
| ENSG00000167995.3 | 310200 | Duchenne muscular dystrophy (3) |
| ENSG00000168036.2 | 116806 | Colorectal cancer (3) |
| ENSG00000168129.1 | 126060 | Anemia, megaloblastic, due to DHFR deficiency (1) (?) |
| ENSG00000168443.3 | 307030 | Glycerol kinase deficiency (3) |
| ENSG00000168447.3 | 600760 | Pseudohypoaldosteronism, type I, 264350 (3) |
| ENSG00000168638.1 | 164790 | Colorectal cancer (3) |
| ENSG00000169031.4 | 120070 | Alport syndrome, autosomal recessive, 203780 (3) |
| ENSG00000169714.3 | 602668 | Myotonic dystrophy 2 (2) |
| ENSG00000169910.3 | 207900 | Argininosuccinicaciduria (3) |
| ENSG00000170266.2 | 230500 | Mucopolysaccharidosis IVB (3) |
| ENSG00000170365.1 | 600794 | Spinal muscular atrophy, distal, with upper limb predominance (2) |
| ENSG00000170370.1 | 600035 | Schizencephaly (3) |
| ENSG00000170624.2 | 601411 | Muscular dystrophy, limb-girdle, type 2F, 601287 (3) |
| ENSG00000170949.4 | 264900 | Factor XI deficiency (3) |
| ENSG00000171365.2 | 300008 | Proteinuria, low molecular weight, with hypercalciuric nephrocalcinosis (3) |
| ENSG00000171444.2 | 159350 | Colorectal cancer (3) |
| ENSG00000171503.1 | 231675 | Glutaricaciduria, type IIC (3) |
| ENSG00000171759.1 | 261600 | [Hyperphenylalaninemia, mild] (3) |
| ENSG00000171862.1 | 601728 | Lhermitte-Duclos syndrome (3) |
| ENSG00000172115.1 | 253270 | Multiple carboxylase deficiency, biotin-responsive (3) |
| ENSG00000172331.2 | 222800 | Hemolytic anemia due to bisphosphoglycerate mutase deficiency (1) |
| ENSG00000172482.1 | 259900 | Hyperoxaluria, primary, type 1 (3) |
| ENSG00000172893.1 | 270400 | Smith-Lemli-Opitz syndrome (2) |
| ENSG00000173599.3 | 266150 | Pyruvate carboxylase deficiency (3) |
| ENSG00000173636.4 | 276000 | Trypsinogen deficiency (1) |
| ENSG00000174231.3 | 600059 | Retinitis pigmentosa-13 (2) |
| ENSG00000174437.2 | 124200 | Darier disease (keratosis follicularis) (2) |
| ENSG00000174775.2 | 190020 | Bladder cancer, 109800 (3) |
| ENSG00000175344.3 | 118511 | Schizophrenia, neurophysiologic defect in (2) |
| ENSG00000175511.3 | 201810 | 3-beta-hydroxysteroid dehydrogenase, type II, deficiency (3) |
| ENSG00000175595.1 | 278760 | Xeroderma pigmentosum, group F (3) |
| ENSG00000176876.2 | 601097 | Neuropathy, recurrent, with pressure palsies, 162500 (3) |
| ENSG00000177000.1 | 236250 | Homocystinuria due to MTHFR deficiency (3) |
| ENSG00000177189.2 | 300075 | Coffin-Lowry syndrome, 303600 (3) |
| ENSG00000177929.3 | 108730 | Brody myopathy, 601003 (3) |
| ENSG00000178053.4 | 601402 | Leukemia, myeloid, acute (1) |
| ENSG00000178537.1 | 212138 | Carnitine-acylcarnitine translocase deficiency (3) |
| ENSG00000178802.3 | 154550 | Carbohydrate-deficient glycoprotein syndrome, type Ib, 602579 (3) |
| ENSG00000179163.3 | 230000 | Fucosidosis (3) |
| ENSG00000179295.2 | 163950 | Noonan syndrome-1 (2) |
| ENSG00000179850.1 | 600354 | Spinal muscular atrophy-3, 253400 (3) |
| ENSG00000180176.2 | 191290 | Segawa syndrome, recessive (3) |
| ENSG00000180398.2 | 601567 | Combined factor V and VIII deficiency, 227300 (3) |
| ENSG00000180616.1 | 182452 | Lung cancer, small cell (3) |
| ENSG00000181163.1 | 164040 | Leukemia, acute promyelocytic, NPM/RARA type (3) |
| ENSG00000181856.3 | 138190 | Diabetes mellitus, noninsulin-dependent (3) |
| ENSG00000182255.2 | 142600 | Hemolytic anemia due to hexokinase deficiency (3) |
| ENSG00000182533.1 | 601253 | Muscular dystrophy, limb-girdle, type IC (3) |
| ENSG00000182636.1 | 602117 | Prader-Willi syndrome (1) (?) |
| ENSG00000183038.2 | 137181 | [Gamma-glutamyltransferase, familial high serum] (2) |
| ENSG00000183072.2 | 600584 | Atrial septal defect with atrioventricular conduction defects, 108900 (3) |
| ENSG00000183770.1 | 110100 | Blepharophimosis, epicanthus inversus, and ptosis, type 1 (2) |
| ENSG00000184156.2 | 602232 | Epilepsy, benign neonatal, type 2, 121201 (3) |
| ENSG00000184315.1 | 300039 | Deafness, X-linked 3, conductive, with stapes fixation, 304400 (3) |
| ENSG00000184486.1 | 300039 | Deafness, X-linked 3, conductive, with stapes fixation, 304400 (3) |
| ENSG00000184627.1 | 210900 | Bloom syndrome (3) |
| ENSG00000185345.3 | 602544 | Parkinson disease, juvenile, type 2, 600116 (3) |
| ENSG00000185630.2 | 176310 | Leukemia, acute pre-B-cell (2) |
| ENSG00000186015.2 | 204200 | Ceroid-lipofuscinosis, neuronal-3, juvenile (3) |
| ENSG00000186051.1 | 186855 | Leukemia-2, T-cell acute lymphoblastic (3) |
