# Supplementary material for: A first-draft human protein-interaction map
Source: Genome Biol. 2004 Aug 13;5(9):R63. doi: 10.1186/gb-2004-5-9-r63 (PMC522870; doi:10.1186/gb-2004-5-9-r63)
Supplement: Additional data file 6 — Gene function predictions for 55 human disease genes [file gb-2004-5-9-r63-s6.doc]

**Lehner and Fraser Supplementary table 5.**

Gene function predictions for 55 human disease genes. The table lists all the disease genes from the core interaction dataset that have 2 or more interaction partners that share a common GO term. All of these GO terms and their accessions are listed. The number of interaction partners associated with each GO term is indicated in brackets for each GO term. The GO predictions are classified according to the approximate probability that the predicted GO term will be associated with the novel gene (see table 3). The predicted Interpro protein domains encoded by the gene are shown for comparison, and in many cases strongly support the predicted functions.

|  |  |  | Predicted GO terms |  |  |
| --- | --- | --- | --- | --- | --- |
| Disease gene | Interpro domains | > 40% probability | 37% probability | 30% probability | 22% probability |
| ENSG00000007372.4, PAIRED BOX PROTEIN PAX-6 (OCULORHOMBIN) (ANIRIDIA, TYPE II PROTEIN). [Source:SWISSPROT;Acc:P26367] | IPR001356 Homeobox, IPR001523 Paired box protein, N-terminal, IPR001356 Homeobox, IPR001523 Paired box protein, N-terminal, IPR001356 Homeobox, IPR001523 Paired box protein, N-terminal |  |  | GO:0003676 (3) nucleic acid binding | GO:0005634 (2) nucleus, GO:0006886 (2) intracellular protein transport, GO:0016020 (2) membrane |
| ENSG00000011052.2, NUCLEOSIDE DIPHOSPHATE KINASE A (EC 2.7.4.6) (NDK A) (NDP KINASE A) (TUMOR METASTATIC PROCESS-ASSOCIATED PROTEIN) (METASTASIS INHIBITION FACTOR NM23) (NM23-H1). [Source:SWISSPROT;Acc:P15531] | IPR001564 Nucleoside diphosphate kinase, IPR001564 Nucleoside diphosphate kinase |  | GO:0005634 (4) nucleus, GO:0005739 (4) mitochondrion | GO:0005524 (3) ATP binding, GO:0005622 (3) intracellular, GO:0005737 (3) cytoplasm, GO:0005840 (3) ribosome, GO:0006412 (3) protein biosynthesis | GO:0003735 (2) structural constituent of ribosome, GO:0003746 (2) translation elongation factor activity, GO:0005525 (2) GTP binding, GO:0005643 (2) nuclear pore, GO:0006414 (2) translational elongation, GO:0006446 (2) regulation of translational initiation, GO:0006607 (2) NLS-bearing substrate-nucleus import, GO:0006886 (2) intracellular protein transport, GO:0008565 (2) protein transporter activity, GO:0016740 (2) transferase activity, GO:0016874 (2) ligase activity |
| ENSG00000015285.2, WISKOTT-ALDRICH SYNDROME PROTEIN (WASP). [Source:SWISSPROT;Acc:P42768] | IPR002965 Proline-rich extensin, IPR001960 WH1, IPR000095 PAK-box/P21-Rho-binding, IPR003124 Actin-binding WH2 |  |  | GO:0005634 (3) nucleus | GO:0003779 (2) actin binding, GO:0005515 (2) protein binding, GO:0006355 (2) regulation of transcription, DNA-dependent, GO:0015629 (2) actin cytoskeleton |
| ENSG00000036473.1, ORNITHINE CARBAMOYLTRANSFERASE, MITOCHONDRIAL PRECURSOR (EC 2.1.3.3) (OTCASE) (ORNITHINE TRANSCARBAMYLASE). [Source:SWISSPROT;Acc:P00480] | IPR006130 Aspartate/ornithine carbamoyltransferase, IPR002292 Ornithine carbamoyltransferase, IPR002082 Aspartate carbamoyltransferase, IPR006131 Aspartate/ornithine carbamoyltransferase, Asp/Orn binding domain, IPR006132 Aspartate/ornithine carbamoyltransferase, carbamoyl-P binding domain |  |  | GO:0016740 (3) transferase activity | GO:0000050 (2) urea cycle, GO:0005737 (2) cytoplasm, GO:0005759 (2) mitochondrial matrix, GO:0006508 (2) proteolysis and peptidolysis, GO:0006526 (2) arginine biosynthesis |
| ENSG00000039068.3, EPITHELIAL-CADHERIN PRECURSOR (E-CADHERIN) (UVOMORULIN) (CADHERIN-1) (CAM 120/80). [Source:SWISSPROT;Acc:P12830] | IPR002126 Cadherin, IPR000233 Cadherin cytoplasmic region, IPR002126 Cadherin, IPR000233 Cadherin cytoplasmic region | GO:0005622 (40) intracellular, GO:0006412 (40) protein biosynthesis, GO:0005840 (27) ribosome, GO:0003735 (24) structural constituent of ribosome, GO:0005842 (12) cytosolic large ribosomal subunit (sensu Eukarya), GO:0005634 (10) nucleus, GO:0005843 (10) cytosolic small ribosomal subunit (sensu Eukarya), GO:0016740 (10) transferase activity, GO:0005739 (9) mitochondrion, GO:0003899 (7) DNA-directed RNA polymerase activity, GO:0003900 (7) DNA-directed RNA polymerase I activity, GO:0003901 (7) DNA-directed RNA polymerase II activity, GO:0003902 (7) DNA-directed RNA polymerase III activity, GO:0006350 (6) transcription, GO:0015934 (5) large ribosomal subunit | GO:0003723 (4) RNA binding, GO:0005762 (4) mitochondrial large ribosomal subunit, GO:0006413 (4) translational initiation, GO:0019843 (4) rRNA binding | GO:0003677 (3) DNA binding, GO:0005524 (3) ATP binding, GO:0005665 (3) DNA-directed RNA polymerase II, core complex, GO:0005737 (3) cytoplasm, GO:0006366 (3) transcription from Pol II promoter, GO:0015935 (3) small ribosomal subunit, GO:0016301 (3) kinase activity, GO:0016787 (3) hydrolase activity | GO:0005730 (2) nucleolus, GO:0006355 (2) regulation of transcription, DNA-dependent, GO:0006445 (2) regulation of translation, GO:0006508 (2) proteolysis and peptidolysis, GO:0008033 (2) tRNA processing, GO:0008151 (2) cell growth and/or maintenance, GO:0016020 (2) membrane |
| ENSG00000049496.1, GLYCINE DEHYDROGENASE [DECARBOXYLATING], MITOCHONDRIAL PRECURSOR (EC 1.4.4.2) (GLYCINE DECARBOXYLASE) (GLYCINE CLEAVAGE SYSTEM P- PROTEIN). [Source:SWISSPROT;Acc:P23378] | IPR002173 Carbohydrate kinase, PfkB, IPR003437 Glycine cleavage system P-protein |  |  |  | GO:0005739 (2) mitochondrion, GO:0005960 (2) glycine cleavage complex, GO:0006546 (2) glycine catabolism |
| ENSG00000060982.3, BRANCHED-CHAIN AMINO ACID AMINOTRANSFERASE, CYTOSOLIC (EC 2.6.1.42) (BCAT(C)) (ECA39 PROTEIN). [Source:SWISSPROT;Acc:P54687] | IPR001544 Aminotransferase, class IV, IPR001544 Aminotransferase, class IV | GO:0005524 (6) ATP binding |  | GO:0005739 (3) mitochondrion | GO:0004222 (2) metalloendopeptidase activity, GO:0006259 (2) DNA metabolism, GO:0006508 (2) proteolysis and peptidolysis, GO:0006633 (2) fatty acid biosynthesis, GO:0016301 (2) kinase activity, GO:0016740 (2) transferase activity |
| ENSG00000063854.1, HYDROXYACYLGLUTATHIONE HYDROLASE (EC 3.1.2.6) (GLYOXALASE II) (GLX II). [Source:SWISSPROT;Acc:Q16775] | IPR001279 Beta-lactamase-like | GO:0008152 (7) metabolism | GO:0005739 (4) mitochondrion | GO:0003824 (3) catalytic activity, GO:0005634 (3) nucleus, GO:0016021 (3) integral to membrane | GO:0005215 (2) transporter activity, GO:0006085 (2) acetyl-CoA biosynthesis, GO:0006810 (2) transport, GO:0008415 (2) acyltransferase activity, GO:0016740 (2) transferase activity, GO:0016787 (2) hydrolase activity |
| ENSG00000069399.1, B-CELL LYMPHOMA 3-ENCODED PROTEIN (BCL-3 PROTEIN). [Source:SWISSPROT;Acc:P20749] | IPR002110 Ankyrin, IPR002965 Proline-rich extensin |  |  | GO:0005634 (3) nucleus, GO:0005737 (3) cytoplasm | GO:0003723 (2) RNA binding, GO:0005871 (2) kinesin complex, GO:0007165 (2) signal transduction |
| ENSG00000075239.2, ACETYL-COA ACETYLTRANSFERASE, MITOCHONDRIAL PRECURSOR (EC 2.3.1.9) (ACETOACETYL-COA THIOLASE) (T2). [Source:SWISSPROT;Acc:P24752] | IPR002155 Thiolase, IPR002155 Thiolase | GO:0008152 (7) metabolism | GO:0005777 (4) peroxisome, GO:0006118 (4) electron transport | GO:0006629 (3) lipid metabolism, GO:0006725 (3) aromatic compound metabolism, GO:0016021 (3) integral to membrane, GO:0016491 (3) oxidoreductase activity | GO:0003824 (2) catalytic activity, GO:0005737 (2) cytoplasm, GO:0005739 (2) mitochondrion, GO:0006631 (2) fatty acid metabolism, GO:0006695 (2) cholesterol biosynthesis, GO:0016740 (2) transferase activity, GO:0016874 (2) ligase activity |
| ENSG00000091140.1, DIHYDROLIPOAMIDE DEHYDROGENASE, MITOCHONDRIAL PRECURSOR (EC 1.8.1.4) (GLYCINE CLEAVAGE SYSTEM L PROTEIN). [Source:SWISSPROT;Acc:P09622] | IPR001100 Pyridine nucleotide-disulphide oxidoreductase, class I, IPR001327 FAD-dependent pyridine nucleotide-disulphide oxidoreductase, IPR000815 Mercuric reductase, IPR000103 Pyridine nucleotide-disulphide oxidoreductase, class-II, IPR004099 Pyridine nucleotide-disulphide oxidoreductase dimerisation domain | GO:0005739 (9) mitochondrion, GO:0006099 (6) tricarboxylic acid cycle, GO:0006096 (5) glycolysis, GO:0008152 (5) metabolism | GO:0016491 (4) oxidoreductase activity | GO:0016874 (3) ligase activity | GO:0006091 (2) energy pathways, GO:0006118 (2) electron transport |
| ENSG00000100243.4, NADH-CYTOCHROME B5 REDUCTASE (EC 1.6.2.2) (B5R). [Source:SWISSPROT;Acc:P00387] | IPR001230 Prenyl group binding site (CAAX box), IPR001834 NADH:cytochrome b5 reductase (CBR), IPR001709 Flavoprotein pyridine nucleotide cytochrome reductase, IPR001221 Phenol hydroxylase reductase, IPR001433 Oxidoreductase FAD/NAD(P)-binding, IPR001230 Prenyl group binding site (CAAX box), IPR001834 NADH:cytochrome b5 reductase (CBR), IPR001709 Flavoprotein pyridine nucleotide cytochrome reductase, IPR001221 Phenol hydroxylase reductase, IPR001433 Oxidoreductase FAD/NAD(P)-binding |  |  | GO:0006207 (3) 'de novo' pyrimidine base biosynthesis, GO:0006221 (3) pyrimidine nucleotide biosynthesis | GO:0005739 (2) mitochondrion |
| ENSG00000100357.2, ADENYLOSUCCINATE LYASE (EC 4.3.2.2) (ADENYLOSUCCINASE) (ASL) (ASASE). [Source:SWISSPROT;Acc:P30566] | IPR000362 Fumarate lyase |  |  | GO:0006118 (3) electron transport, GO:0006725 (3) aromatic compound metabolism, GO:0016740 (3) transferase activity | GO:0008415 (2) acyltransferase activity |
| ENSG00000102081.2, FRAGILE X MENTAL RETARDATION 1 PROTEIN (PROTEIN FMR-1) (FMRP). [Source:SWISSPROT;Acc:Q06787] | IPR004087 KH domain, IPR004088 KH domain, type 1 |  |  | GO:0005634 (3) nucleus | GO:0003702 (2) RNA polymerase II transcription factor activity, GO:0005737 (2) cytoplasm, GO:0006355 (2) regulation of transcription, DNA-dependent, GO:0007275 (2) development |
| ENSG00000102144.2, PHOSPHOGLYCERATE KINASE 1 (EC 2.7.2.3) (PRIMER RECOGNITION PROTEIN 2) (PRP 2). [Source:SWISSPROT;Acc:P00558] | IPR001576 Phosphoglycerate kinase |  |  | GO:0006096 (3) glycolysis | GO:0005524 (2) ATP binding, GO:0005737 (2) cytoplasm |
| ENSG00000102314.2, 40S RIBOSOMAL PROTEIN S4, X ISOFORM (SINGLE COPY ABUNDANT MRNA PROTEIN) (SCR10). [Source:SWISSPROT;Acc:P12750] | IPR000876 Ribosomal protein S4E, IPR005824 KOW, IPR002942 RNA-binding S4 | GO:0005622 (22) intracellular, GO:0006412 (22) protein biosynthesis, GO:0005840 (16) ribosome, GO:0003735 (15) structural constituent of ribosome, GO:0005842 (9) cytosolic large ribosomal subunit (sensu Eukarya), GO:0005843 (5) cytosolic small ribosomal subunit (sensu Eukarya) |  | GO:0003723 (3) RNA binding, GO:0005730 (3) nucleolus, GO:0006413 (3) translational initiation, GO:0015934 (3) large ribosomal subunit, GO:0016020 (3) membrane | GO:0005737 (2) cytoplasm, GO:0006364 (2) rRNA processing, GO:0008033 (2) tRNA processing, GO:0008151 (2) cell growth and/or maintenance, GO:0016021 (2) integral to membrane |
| ENSG00000104313.2, EYES ABSENT HOMOLOG 1. [Source:SWISSPROT;Acc:Q99502] | IPR005834 Haloacid dehalogenase-like hydrolase, IPR005834 Haloacid dehalogenase-like hydrolase | GO:0005634 (12) nucleus, GO:0003676 (7) nucleic acid binding, GO:0006355 (6) regulation of transcription, DNA-dependent |  | GO:0007345 (3) embryogenesis and morphogenesis | GO:0003700 (2) transcription factor activity, GO:0003713 (2) transcription co-activator activity, GO:0003723 (2) RNA binding, GO:0005524 (2) ATP binding, GO:0005643 (2) nuclear pore, GO:0005654 (2) nucleoplasm, GO:0005737 (2) cytoplasm, GO:0006886 (2) intracellular protein transport, GO:0007275 (2) development, GO:0008151 (2) cell growth and/or maintenance |
| ENSG00000104687.2, GLUTATHIONE REDUCTASE, MITOCHONDRIAL PRECURSOR (EC 1.8.1.7) (GR) (GRASE). [Source:SWISSPROT;Acc:P00390] | IPR001100 Pyridine nucleotide-disulphide oxidoreductase, class I, IPR001327 FAD-dependent pyridine nucleotide-disulphide oxidoreductase, IPR000815 Mercuric reductase, IPR000103 Pyridine nucleotide-disulphide oxidoreductase, class-II, IPR004099 Pyridine nucleotide-disulphide oxidoreductase dimerisation domain | GO:0005739 (8) mitochondrion, GO:0006096 (5) glycolysis, GO:0006099 (5) tricarboxylic acid cycle | GO:0008152 (4) metabolism, GO:0016491 (4) oxidoreductase activity | GO:0016874 (3) ligase activity | GO:0006118 (2) electron transport |
| ENSG00000104884.2, TFIIH BASAL TRANSCRIPTION FACTOR COMPLEX HELICASE SUBUNIT (EC 3.6.1.-) (DNA-REPAIR PROTEIN COMPLEMENTING XP-D CELLS) (XERODERMA PIGMENTOSUM GROUP D COMPLEMENTING PROTEIN) (CXPD) (DNA EXCISION REPAIR PROTEIN ERCC-2). [Source:SWISSPROT;Acc:P18074] | IPR001687 ATP/GTP-binding site motif A (P-loop), IPR002464 ATP-dependent helicase, DEAH-box, IPR001945 Xeroderma pigmentosum group D protein (XPD) |  |  | GO:0005524 (3) ATP binding, GO:0005625 (3) soluble fraction, GO:0005737 (3) cytoplasm, GO:0006422 (3) aspartyl-tRNA aminoacylation, GO:0008152 (3) metabolism, GO:0016874 (3) ligase activity | GO:0004815 (2) aspartate-tRNA ligase activity, GO:0009058 (2) biosynthesis, GO:0016740 (2) transferase activity, GO:0016847 (2) 1-aminocyclopropane-1-carboxylate synthase activity |
| ENSG00000105379.1, ELECTRON TRANSFER FLAVOPROTEIN BETA-SUBUNIT (BETA-ETF). [Source:SWISSPROT;Acc:P38117] | IPR000049 Electron transfer flavoprotein beta-subunit | GO:0008152 (6) metabolism |  |  | GO:0003824 (2) catalytic activity, GO:0005634 (2) nucleus, GO:0005739 (2) mitochondrion, GO:0006118 (2) electron transport, GO:0016021 (2) integral to membrane |
| ENSG00000105552.4, BRANCHED-CHAIN AMINO ACID AMINOTRANSFERASE, MITOCHONDRIAL PRECURSOR (EC 2.6.1.42) (BCAT(M)) (PLACENTAL PROTEIN 18) (PP18). [Source:SWISSPROT;Acc:O15382] | IPR001544 Aminotransferase, class IV, IPR001544 Aminotransferase, class IV | GO:0005524 (6) ATP binding |  | GO:0005739 (3) mitochondrion | GO:0004222 (2) metalloendopeptidase activity, GO:0006259 (2) DNA metabolism, GO:0006508 (2) proteolysis and peptidolysis, GO:0006633 (2) fatty acid biosynthesis, GO:0016301 (2) kinase activity, GO:0016740 (2) transferase activity |
| ENSG00000105618.3, PRE-MRNA PROCESSING FACTOR 31 HOMOLOG; PRE-MRNA PROCESSING FACTOR 31 HOMOLOG (YEAST). [Source:RefSeq;Acc:NM_015629] | IPR002687 Pre-mRNA processing ribonucleoprotein, binding region, IPR002687 Pre-mRNA processing ribonucleoprotein, binding region | GO:0006371 (8) mRNA splicing, GO:0005681 (6) spliceosome complex, GO:0008248 (5) pre-mRNA splicing factor activity |  | GO:0005634 (3) nucleus, GO:0030529 (3) ribonucleoprotein complex | GO:0030532 (2) small nuclear ribonucleoprotein complex |
| ENSG00000105641.1, SODIUM/IODIDE COTRANSPORTER (NA(+)/I(-) COTRANSPORTER) (SODIUM-IODIDE SYMPORTER) (NA+/I-SYMPORTER). [Source:SWISSPROT;Acc:Q92911] | IPR001734 Na+/solute symporter |  |  | GO:0005524 (3) ATP binding, GO:0005739 (3) mitochondrion | GO:0004527 (2) exonuclease activity, GO:0005622 (2) intracellular, GO:0008152 (2) metabolism, GO:0009374 (2) biotin binding, GO:0016874 (2) ligase activity |
| ENSG00000105953.3, 2-OXOGLUTARATE DEHYDROGENASE E1 COMPONENT, MITOCHONDRIAL PRECURSOR (EC 1.2.4.2) (ALPHA-KETOGLUTARATE DEHYDROGENASE). [Source:SWISSPROT;Acc:Q02218] | IPR001017 Dehydrogenase, E1 component, IPR005475 Transketolase, central region, IPR001017 Dehydrogenase, E1 component, IPR005475 Transketolase, central region | GO:0005739 (8) mitochondrion, GO:0006099 (6) tricarboxylic acid cycle, GO:0006118 (5) electron transport | GO:0006096 (4) glycolysis, GO:0008152 (4) metabolism | GO:0015036 (3) disulfide oxidoreductase activity | GO:0006085 (2) acetyl-CoA biosynthesis, GO:0006091 (2) energy pathways, GO:0008177 (2) succinate dehydrogenase (ubiquinone) activity, GO:0008415 (2) acyltransferase activity, GO:0009060 (2) aerobic respiration, GO:0016740 (2) transferase activity, GO:0016874 (2) ligase activity, GO:0019737 (2) quinol:fumarate oxidoreductase activity |
| ENSG00000106588.1, PROTEASOME SUBUNIT ALPHA TYPE 2 (EC 3.4.25.1) (PROTEASOME COMPONENT C3) (MACROPAIN SUBUNIT C3) (MULTICATALYTIC ENDOPEPTIDASE COMPLEX SUBUNIT C3). [Source:SWISSPROT;Acc:P25787] | IPR000426 Proteasome subunit, A-type, IPR001353 Multispecific proteasome protease | GO:0005829 (12) cytosol, GO:0005837 (11) 26S proteasome, GO:0004299 (10) proteasome endopeptidase activity, GO:0005839 (10) proteasome core complex (sensu Eukarya), GO:0006511 (10) ubiquitin-dependent protein catabolism |  | GO:0004175 (3) endopeptidase activity, GO:0005524 (3) ATP binding, GO:0005634 (3) nucleus, GO:0016787 (3) hydrolase activity | GO:0006508 (2) proteolysis and peptidolysis, GO:0030163 (2) protein catabolism |
| ENSG00000110921.2, MEVALONATE KINASE (EC 2.7.1.36) (MK). [Source:SWISSPROT;Acc:Q03426] | IPR006203 GHMP kinase, ATP-binding region, IPR006206 Mevalonate and galactokinase, IPR001174 Galactokinase/homoserine kinase, IPR006204 GHMP kinase | GO:0005622 (10) intracellular, GO:0006412 (10) protein biosynthesis, GO:0016740 (6) transferase activity, GO:0003735 (5) structural constituent of ribosome, GO:0005840 (5) ribosome | GO:0003900 (4) DNA-directed RNA polymerase I activity, GO:0003901 (4) DNA-directed RNA polymerase II activity, GO:0003902 (4) DNA-directed RNA polymerase III activity, GO:0005634 (4) nucleus, GO:0005843 (4) cytosolic small ribosomal subunit (sensu Eukarya), GO:0008299 (4) isoprenoid biosynthesis | GO:0003899 (3) DNA-directed RNA polymerase activity, GO:0005665 (3) DNA-directed RNA polymerase II, core complex, GO:0005739 (3) mitochondrion, GO:0006350 (3) transcription, GO:0006366 (3) transcription from Pol II promoter, GO:0015935 (3) small ribosomal subunit | GO:0003677 (2) DNA binding, GO:0004337 (2) geranyltranstransferase activity, GO:0006118 (2) electron transport, GO:0006695 (2) cholesterol biosynthesis |
| ENSG00000111669.1, TRIOSEPHOSPHATE ISOMERASE (EC 5.3.1.1) (TIM). [Source:SWISSPROT;Acc:P00938] | IPR000652 Triosephosphate isomerase |  |  | GO:0006096 (3) glycolysis | GO:0005829 (2) cytosol, GO:0016740 (2) transferase activity |
| ENSG00000112319.4, EYES ABSENT HOMOLOG 4. [Source:SWISSPROT;Acc:O95677] | IPR005834 Haloacid dehalogenase-like hydrolase, IPR005834 Haloacid dehalogenase-like hydrolase, IPR005834 Haloacid dehalogenase-like hydrolase | GO:0005634 (12) nucleus, GO:0003676 (7) nucleic acid binding, GO:0006355 (6) regulation of transcription, DNA-dependent |  | GO:0007345 (3) embryogenesis and morphogenesis | GO:0003700 (2) transcription factor activity, GO:0003713 (2) transcription co-activator activity, GO:0003723 (2) RNA binding, GO:0005524 (2) ATP binding, GO:0005643 (2) nuclear pore, GO:0005654 (2) nucleoplasm, GO:0005737 (2) cytoplasm, GO:0006886 (2) intracellular protein transport, GO:0007275 (2) development, GO:0008151 (2) cell growth and/or maintenance |
| ENSG00000114491.2, URIDINE 5'-MONOPHOSPHATE SYNTHASE (UMP SYNTHASE) [INCLUDES: OROTATE PHOSPHORIBOSYLTRANSFERASE (EC 2.4.2.10) (OPRTASE); OROTIDINE 5'- PHOSPHATE DECARBOXYLASE (EC 4.1.1.23) (OMPDECASE)]. [Source:SWISSPROT;Acc:P11172] | IPR002375 Purine/pyrimidine phosphoribosyl transferase, IPR001754 Orotidine 5'-phosphate decarboxylase, IPR000836 Phosphoribosyltransferase |  | GO:0016020 (4) membrane | GO:0003735 (3) structural constituent of ribosome, GO:0005622 (3) intracellular, GO:0005737 (3) cytoplasm, GO:0005840 (3) ribosome, GO:0006412 (3) protein biosynthesis, GO:0006413 (3) translational initiation, GO:0016021 (3) integral to membrane, GO:0016491 (3) oxidoreductase activity | GO:0005634 (2) nucleus, GO:0005739 (2) mitochondrion, GO:0005783 (2) endoplasmic reticulum, GO:0005843 (2) cytosolic small ribosomal subunit (sensu Eukarya), GO:0005887 (2) integral to plasma membrane, GO:0006118 (2) electron transport, GO:0006207 (2) 'de novo' pyrimidine base biosynthesis, GO:0006221 (2) pyrimidine nucleotide biosynthesis, GO:0008151 (2) cell growth and/or maintenance |
| ENSG00000117118.1, SUCCINATE DEHYDROGENASE [UBIQUINONE] IRON-SULFUR PROTEIN, MITOCHONDRIAL PRECURSOR (EC 1.3.5.1) (IP) (IRON-SULFUR SUBUNIT OF COMPLEX II). [Source:SWISSPROT;Acc:P21912] | IPR006058 2Fe-2S ferredoxin, IPR001450 4Fe-4S ferredoxin, iron-sulfur binding domain, IPR001041 Ferredoxin | GO:0005739 (6) mitochondrion | GO:0006118 (4) electron transport | GO:0006091 (3) energy pathways, GO:0006096 (3) glycolysis, GO:0006099 (3) tricarboxylic acid cycle, GO:0008152 (3) metabolism, GO:0015036 (3) disulfide oxidoreductase activity | GO:0006085 (2) acetyl-CoA biosynthesis, GO:0008415 (2) acyltransferase activity, GO:0016740 (2) transferase activity |
| ENSG00000117308.2, UDP-GLUCOSE 4-EPIMERASE (EC 5.1.3.2) (GALACTOWALDENASE) (UDP- GALACTOSE 4-EPIMERASE). [Source:SWISSPROT;Acc:Q14376] | IPR001509 NAD-dependent epimerase/dehydratase |  |  | GO:0016740 (3) transferase activity | GO:0005524 (2) ATP binding, GO:0005737 (2) cytoplasm, GO:0005783 (2) endoplasmic reticulum, GO:0005975 (2) carbohydrate metabolism, GO:0006012 (2) galactose metabolism, GO:0006486 (2) protein amino acid glycosylation, GO:0016301 (2) kinase activity, GO:0016757 (2) transferase activity, transferring glycosyl groups |
| ENSG00000119508.4, NUCLEAR HORMONE RECEPTOR NOR-1 (NEURON-DERIVED ORPHAN RECEPTOR 1) (MITOGEN INDUCED NUCLEAR ORPHAN RECEPTOR). [Source:SWISSPROT;Acc:Q92570] | IPR001628 Zn-finger, C4-type steroid receptor, IPR003072 Orphan nuclear receptor, NOR1 type, IPR003070 Orphan nuclear receptor, IPR001723 Steroid hormone receptor, IPR000324 Vitamin D receptor, IPR003078 Retinoic acid receptor, IPR000536 Ligand-binding domain of nuclear hormone receptor, IPR001628 Zn-finger, C4-type steroid receptor, IPR003072 Orphan nuclear receptor, NOR1 type, IPR003070 Orphan nuclear receptor, IPR001723 Steroid hormone receptor, IPR000324 Vitamin D receptor, IPR003078 Retinoic acid receptor, IPR000536 Ligand-binding domain of nuclear hormone receptor | GO:0005837 (6) 26S proteasome, GO:0005829 (5) cytosol | GO:0005524 (4) ATP binding |  | GO:0004299 (2) proteasome endopeptidase activity, GO:0004713 (2) protein-tyrosine kinase activity, GO:0005634 (2) nucleus, GO:0005838 (2) proteasome regulatory particle (sensu Eukarya), GO:0005839 (2) proteasome core complex (sensu Eukarya), GO:0006468 (2) protein amino acid phosphorylation, GO:0006511 (2) ubiquitin-dependent protein catabolism, GO:0008575 (2) proteasome ATPase activity, GO:0016740 (2) transferase activity, GO:0016787 (2) hydrolase activity |
| ENSG00000125814.4, BETA-SOLUBLE NSF ATTACHMENT PROTEIN (SNAP-BETA) (N-ETHYLMALEIMIDE- SENSITIVE FACTOR ATTACHMENT PROTEIN, BETA). [Source:SWISSPROT;Acc:Q9H115] | IPR000744 NSF attachment protein |  |  | GO:0005871 (3) kinesin complex, GO:0006886 (3) intracellular protein transport, GO:0008565 (3) protein transporter activity | GO:0006887 (2) exocytosis, GO:0016021 (2) integral to membrane |
| ENSG00000129084.3, PROTEASOME SUBUNIT ALPHA TYPE 1 (EC 3.4.25.1) (PROTEASOME COMPONENT C2) (MACROPAIN SUBUNIT C2) (MULTICATALYTIC ENDOPEPTIDASE COMPLEX SUBUNIT C2) (PROTEASOME NU CHAIN) (30 KDA PROSOMAL PROTEIN) (PROS-30). [Source:SWISSPROT;Acc:P25786] | IPR000426 Proteasome subunit, A-type, IPR000515 Binding-protein-dependent transport systems inner membrane component, IPR001353 Multispecific proteasome protease, IPR000426 Proteasome subunit, A-type, IPR000515 Binding-protein-dependent transport systems inner membrane component, IPR001353 Multispecific proteasome protease | GO:0005837 (20) 26S proteasome, GO:0005829 (19) cytosol, GO:0004299 (11) proteasome endopeptidase activity, GO:0005839 (11) proteasome core complex (sensu Eukarya), GO:0006511 (11) ubiquitin-dependent protein catabolism, GO:0005524 (5) ATP binding, GO:0005634 (5) nucleus, GO:0006508 (5) proteolysis and peptidolysis, GO:0016787 (5) hydrolase activity |  | GO:0008575 (3) proteasome ATPase activity, GO:0030163 (3) protein catabolism | GO:0004175 (2) endopeptidase activity, GO:0005838 (2) proteasome regulatory particle (sensu Eukarya), GO:0006413 (2) translational initiation, GO:0006955 (2) immune response |
| ENSG00000130707.2, ARGININOSUCCINATE SYNTHASE (EC 6.3.4.5) (CITRULLINE--ASPARTATE LIGASE). [Source:SWISSPROT;Acc:P00966] | IPR001518 Argininosuccinate synthase |  |  |  | GO:0000050 (2) urea cycle, GO:0006526 (2) arginine biosynthesis, GO:0016740 (2) transferase activity |
| ENSG00000130826.2, DYSKERIN (NUCLEOLAR PROTEIN NAP57) (CBF5 HOMOLOG). [Source:SWISSPROT;Acc:O60832] | IPR002478 PUA domain, IPR002501 Pseudouridylate synthase TruB, N-terminal | GO:0006412 (15) protein biosynthesis, GO:0005622 (14) intracellular, GO:0005840 (10) ribosome, GO:0003735 (7) structural constituent of ribosome, GO:0005843 (6) cytosolic small ribosomal subunit (sensu Eukarya) | GO:0005739 (4) mitochondrion, GO:0005842 (4) cytosolic large ribosomal subunit (sensu Eukarya) | GO:0003723 (3) RNA binding, GO:0006364 (3) rRNA processing, GO:0006446 (3) regulation of translational initiation, GO:0015935 (3) small ribosomal subunit | GO:0003743 (2) translation initiation factor activity, GO:0003746 (2) translation elongation factor activity, GO:0005524 (2) ATP binding, GO:0005525 (2) GTP binding, GO:0005634 (2) nucleus, GO:0005730 (2) nucleolus, GO:0006414 (2) translational elongation, GO:0008304 (2) eukaryotic translation initiation factor 4 complex, GO:0016787 (2) hydrolase activity, GO:0019843 (2) rRNA binding |
| ENSG00000131828.2, PYRUVATE DEHYDROGENASE E1 COMPONENT ALPHA SUBUNIT, SOMATIC FORM, MITOCHONDRIAL PRECURSOR (EC 1.2.4.1) (PDHE1-A TYPE I). [Source:SWISSPROT;Acc:P08559] | IPR001017 Dehydrogenase, E1 component | GO:0005739 (7) mitochondrion |  | GO:0006096 (3) glycolysis | GO:0006085 (2) acetyl-CoA biosynthesis, GO:0006091 (2) energy pathways, GO:0006099 (2) tricarboxylic acid cycle, GO:0006118 (2) electron transport, GO:0008152 (2) metabolism, GO:0008415 (2) acyltransferase activity, GO:0015036 (2) disulfide oxidoreductase activity, GO:0016491 (2) oxidoreductase activity, GO:0016740 (2) transferase activity |
| ENSG00000131979.2, GTP CYCLOHYDROLASE I (EC 3.5.4.16) (GTP-CH-I). [Source:SWISSPROT;Acc:P30793] | IPR001474 GTP cyclohydrolase I, IPR001474 GTP cyclohydrolase I |  |  | GO:0008299 (3) isoprenoid biosynthesis | GO:0004337 (2) geranyltranstransferase activity, GO:0006730 (2) one-carbon compound metabolism, GO:0016740 (2) transferase activity |
| ENSG00000132142.4, ACETYL-COA CARBOXYLASE 1 (EC 6.4.1.2) (ACC-ALPHA) [INCLUDES: BIOTIN CARBOXYLASE (EC 6.3.4.14)]. [Source:SWISSPROT;Acc:Q13085] | IPR001882 Biotin-requiring enzyme, attachment site, IPR005479 Carbamoyl-phosphate synthase L chain, ATP-binding, IPR005481 Carbamoyl-phosphate synthetase large chain, N-terminal, IPR000089 Biotin/lipoyl attachment, IPR000022 Carboxyl transferase, IPR005482 Biotin carboxylase, C-terminal | GO:0008152 (9) metabolism, GO:0005739 (6) mitochondrion | GO:0016491 (4) oxidoreductase activity, GO:0016787 (4) hydrolase activity | GO:0003824 (3) catalytic activity, GO:0005524 (3) ATP binding, GO:0005622 (3) intracellular, GO:0005777 (3) peroxisome, GO:0006412 (3) protein biosynthesis, GO:0016021 (3) integral to membrane, GO:0016740 (3) transferase activity, GO:0016874 (3) ligase activity | GO:0005624 (2) membrane fraction, GO:0005737 (2) cytoplasm, GO:0005783 (2) endoplasmic reticulum, GO:0005887 (2) integral to plasma membrane, GO:0006006 (2) glucose metabolism, GO:0006099 (2) tricarboxylic acid cycle, GO:0006118 (2) electron transport, GO:0006508 (2) proteolysis and peptidolysis, GO:0006629 (2) lipid metabolism, GO:0006631 (2) fatty acid metabolism, GO:0006633 (2) fatty acid biosynthesis, GO:0006810 (2) transport, GO:0007165 (2) signal transduction, GO:0008610 (2) lipid biosynthesis, GO:0009374 (2) biotin binding, GO:0015935 (2) small ribosomal subunit, GO:0030145 (2) manganese ion binding |
| ENSG00000133835.1, ESTRADIOL 17 BETA-DEHYDROGENASE 4 (EC 1.1.1.62) (17-BETA-HSD 4) (17-BETA-HYDROXYSTEROID DEHYDROGENASE 4). [Source:SWISSPROT;Acc:P51659] | IPR002198 Short-chain dehydrogenase/reductase SDR, IPR002347 Glucose/ribitol dehydrogenase, IPR003560 2,3-dihydro-2,3-dihydroxybenzoate dehydrogenase, IPR002539 MaoC-like dehydratase, IPR003033 Sterol-binding |  |  | GO:0008152 (3) metabolism | GO:0005777 (2) peroxisome, GO:0006091 (2) energy pathways, GO:0006631 (2) fatty acid metabolism, GO:0008415 (2) acyltransferase activity, GO:0016740 (2) transferase activity |
| ENSG00000134899.1, DNA-REPAIR PROTEIN COMPLEMENTING XP-G CELLS (XERODERMA PIGMENTOSUM GROUP G COMPLEMENTING PROTEIN) (DNA EXCISION REPAIR PROTEIN ERCC-5). [Source:SWISSPROT;Acc:P28715] | IPR006085 XPG N-terminal, IPR000513 5'3'-Exonuclease N- and I-domain, IPR006085 XPG N-terminal, IPR006086 XPG I domain, IPR001044 Xeroderma pigmentosum group G protein, IPR006084 DNA repair protein (XPGC)/yeast Rad, IPR000513 5'3'-Exonuclease N- and I-domain |  |  |  | GO:0005634 (2) nucleus, GO:0005840 (2) ribosome, GO:0006412 (2) protein biosynthesis, GO:0006950 (2) response to stress |
| ENSG00000136997.1, MYC PROTO-ONCOGENE PROTEIN (C-MYC). [Source:SWISSPROT;Acc:P01106] | IPR001092 Basic helix-loop-helix dimerization domain bHLH, IPR002418 Transcription regulator Myc, IPR003327 Leucine zipper, Myc |  |  |  | GO:0003713 (2) transcription co-activator activity, GO:0005634 (2) nucleus, GO:0006355 (2) regulation of transcription, DNA-dependent, GO:0006366 (2) transcription from Pol II promoter |
| ENSG00000139197.1, PEROXISOMAL TARGETING SIGNAL 1 RECEPTOR (PEROXISMORE RECEPTOR 1) (PEROXISOMAL C-TERMINAL TARGETING SIGNAL IMPORT RECEPTOR) (PTS1-BP) (PEROXIN-5) (PTS1 RECEPTOR). [Source:SWISSPROT;Acc:P50542] | IPR001440 TPR repeat, IPR001440 TPR repeat | GO:0005777 (5) peroxisome |  | GO:0005634 (3) nucleus, GO:0005739 (3) mitochondrion, GO:0006118 (3) electron transport, GO:0006810 (3) transport | GO:0006631 (2) fatty acid metabolism |
| ENSG00000140374.3, ELECTRON TRANSFER FLAVOPROTEIN ALPHA-SUBUNIT, MITOCHONDRIAL PRECURSOR (ALPHA-ETF). [Source:SWISSPROT;Acc:P13804] | IPR001308 Electron transfer flavoprotein, alpha subunit | GO:0008152 (6) metabolism |  |  | GO:0003824 (2) catalytic activity, GO:0005634 (2) nucleus, GO:0005739 (2) mitochondrion, GO:0006118 (2) electron transport, GO:0016021 (2) integral to membrane |
| ENSG00000141959.3, 6-PHOSPHOFRUCTOKINASE, LIVER TYPE (EC 2.7.1.11) (PHOSPHOFRUCTOKINASE 1) (PHOSPHOHEXOKINASE) (PHOSPHOFRUCTO-1-KINASE ISOZYME B) (PFK-B). [Source:SWISSPROT;Acc:P17858] | IPR000023 Phosphofructokinase, IPR000023 Phosphofructokinase |  |  | GO:0016740 (3) transferase activity | GO:0005634 (2) nucleus, GO:0006096 (2) glycolysis, GO:0006414 (2) translational elongation |
| ENSG00000144231.1, DNA-DIRECTED RNA POLYMERASE II 16 KDA POLYPEPTIDE (EC 2.7.7.6) (RPB4). [Source:SWISSPROT;Acc:O15514] | IPR005574 RNA polymerase Rpb4 | GO:0005634 (5) nucleus | GO:0003899 (4) DNA-directed RNA polymerase activity, GO:0003900 (4) DNA-directed RNA polymerase I activity, GO:0003901 (4) DNA-directed RNA polymerase II activity, GO:0003902 (4) DNA-directed RNA polymerase III activity, GO:0005665 (4) DNA-directed RNA polymerase II, core complex, GO:0006350 (4) transcription, GO:0006366 (4) transcription from Pol II promoter, GO:0016740 (4) transferase activity |  |  |
| ENSG00000148672.1, GLUTAMATE DEHYDROGENASE 1, MITOCHONDRIAL PRECURSOR (EC 1.4.1.3) (GDH). [Source:SWISSPROT;Acc:P00367] | IPR006095 Glu/Leu/Phe/Val dehydrogenase, IPR006096 Glu/Leu/Phe/Val dehydrogenase, C terminal, IPR006097 Glu/Leu/Phe/Val dehydrogenase, dimerisation domain |  | GO:0005739 (4) mitochondrion | GO:0006096 (3) glycolysis, GO:0008152 (3) metabolism | GO:0006085 (2) acetyl-CoA biosynthesis, GO:0006099 (2) tricarboxylic acid cycle, GO:0008415 (2) acyltransferase activity, GO:0016491 (2) oxidoreductase activity, GO:0016740 (2) transferase activity |
| ENSG00000149397.2, PORPHOBILINOGEN DEAMINASE (EC 4.3.1.8) (HYDROXYMETHYLBILANE SYNTHASE) (HMBS) (PRE-UROPORPHYRINOGEN SYNTHASE) (PBG-D). [Source:SWISSPROT;Acc:P08397] | IPR000860 Porphobilinogen deaminase |  |  | GO:0008299 (3) isoprenoid biosynthesis | GO:0004337 (2) geranyltranstransferase activity, GO:0005524 (2) ATP binding, GO:0006433 (2) prolyl-tRNA aminoacylation, GO:0016740 (2) transferase activity |
| ENSG00000151224.1, S-ADENOSYLMETHIONINE SYNTHETASE ALPHA AND BETA FORMS (EC 2.5.1.6) (METHIONINE ADENOSYLTRANSFERASE) (ADOMET SYNTHETASE) (MAT-I/III). [Source:SWISSPROT;Acc:Q00266] | IPR002133 S-adenosylmethionine synthetase |  | GO:0005634 (4) nucleus, GO:0006508 (4) proteolysis and peptidolysis | GO:0016740 (3) transferase activity | GO:0000059 (2) protein-nucleus import, docking, GO:0003735 (2) structural constituent of ribosome, GO:0005524 (2) ATP binding, GO:0005622 (2) intracellular, GO:0005643 (2) nuclear pore, GO:0005737 (2) cytoplasm, GO:0005840 (2) ribosome, GO:0006183 (2) GTP biosynthesis, GO:0006412 (2) protein biosynthesis, GO:0008450 (2) O-sialoglycoprotein endopeptidase activity, GO:0016301 (2) kinase activity |
| ENSG00000152556.3, 6-PHOSPHOFRUCTOKINASE, MUSCLE TYPE (EC 2.7.1.11) (PHOSPHOFRUCTOKINASE 1) (PHOSPHOHEXOKINASE) (PHOSPHOFRUCTO-1-KINASE ISOZYME A) (PFK-A) (PHOSPHOFRUCTOKINASE-M). [Source:SWISSPROT;Acc:P08237] | IPR000023 Phosphofructokinase |  |  | GO:0016740 (3) transferase activity | GO:0000287 (2) magnesium ion binding, GO:0005634 (2) nucleus, GO:0006002 (2) fructose 6-phosphate metabolism, GO:0006096 (2) glycolysis, GO:0006414 (2) translational elongation |
| ENSG00000158581.3, ADENINE PHOSPHORIBOSYLTRANSFERASE (EC 2.4.2.7) (APRT). [Source:SWISSPROT;Acc:P07741] | IPR002375 Purine/pyrimidine phosphoribosyl transferase, IPR000836 Phosphoribosyltransferase, IPR000694 Proline-rich region |  | GO:0005739 (4) mitochondrion | GO:0008152 (3) metabolism | GO:0006085 (2) acetyl-CoA biosynthesis, GO:0006091 (2) energy pathways, GO:0006096 (2) glycolysis, GO:0006118 (2) electron transport, GO:0008415 (2) acyltransferase activity, GO:0015036 (2) disulfide oxidoreductase activity, GO:0016740 (2) transferase activity |
| ENSG00000159267.4, BIOTIN--PROTEIN LIGASE (EC 6.3.4.-) (BIOTIN APO-PROTEIN LIGASE) [INCLUDES: BIOTIN--[METHYLMALONYL-COA-CARBOXYLTRANSFERASE] LIGASE (EC 6.3.4.9); BIOTIN--[PROPIONYL-COA-CARBOXYLASE [ATP-HYDROLYZING]] LIGASE (EC 6.3.4.10) (HOLOCARBOXYLASE SYNTHETASE) (HCS); BIOTIN--[METHYLCROTONOYL-COA-CARBOXYLASE] LIGASE (EC 6.3.4.11); BIOTIN--[ACETYL-COA-CARBOXYLASE] LIGASE (EC 6.3.4.15)]. [Source:SWISSPROT;Acc:P50747] | IPR003142 Biotin protein ligase, C-terminal, IPR004143 Biotin/lipoate A/B protein ligase domain, IPR003142 Biotin protein ligase, C-terminal, IPR004143 Biotin/lipoate A/B protein ligase domain, IPR007087 Zn-finger, C2H2 type | GO:0005739 (6) mitochondrion, GO:0005215 (5) transporter activity, GO:0005753 (5) proton-transporting ATP synthase complex (sensu Eukarya), GO:0006754 (5) ATP biosynthesis, GO:0015992 (5) proton transport, GO:0016787 (5) hydrolase activity | GO:0005624 (4) membrane fraction, GO:0015078 (4) hydrogen ion transporter activity |  | GO:0003936 (2) hydrogen-transporting two-sector ATPase activity, GO:0005224 (2) ATP-binding and phosphorylation-dependent chloride channel activity, GO:0005524 (2) ATP binding, GO:0006091 (2) energy pathways |
| ENSG00000160200.4, CYSTATHIONINE BETA-SYNTHASE (EC 4.2.1.22) (SERINE SULFHYDRASE) (BETA-THIONASE). [Source:SWISSPROT;Acc:P35520] | IPR001216 Cysteine synthase/cystathionine beta-synthase P-phosphate attachment site, IPR001926 Pyridoxal-5'-phosphate-dependent enzyme, beta family, IPR000644 CBS domain, IPR001472 Bipartite nuclear localization signal, IPR001216 Cysteine synthase/cystathionine beta-synthase P-phosphate attachment site, IPR001926 Pyridoxal-5'-phosphate-dependent enzyme, beta family, IPR000644 CBS domain, IPR001472 Bipartite nuclear localization signal |  |  |  | GO:0005783 (2) endoplasmic reticulum, GO:0006486 (2) protein amino acid glycosylation, GO:0016021 (2) integral to membrane, GO:0016757 (2) transferase activity, transferring glycosyl groups |
| ENSG00000162374.3, ELAV-LIKE PROTEIN 4 (PARANEOPLASTIC ENCEPHALOMYELITIS ANTIGEN HUD) (HU-ANTIGEN D). [Source:SWISSPROT;Acc:P26378] | IPR000504 RNA-binding region RNP-1 (RNA recognition motif), IPR002343 Paraneoplastic encephalomyelitis antigen, IPR000504 RNA-binding region RNP-1 (RNA recognition motif), IPR002343 Paraneoplastic encephalomyelitis antigen |  |  | GO:0003676 (3) nucleic acid binding, GO:0005634 (3) nucleus | GO:0003723 (2) RNA binding, GO:0005737 (2) cytoplasm, GO:0006396 (2) RNA processing, GO:0006886 (2) intracellular protein transport |
| ENSG00000164025.4, ALCOHOL DEHYDROGENASE CLASS III CHI CHAIN (EC 1.1.1.1) (GLUTATHIONE- DEPENDENT FORMALDEHYDE DEHYDROGENASE) (EC 1.2.1.1) (FDH). [Source:SWISSPROT;Acc:P11766] | IPR002328 Zinc-containing alcohol dehydrogenase, IPR002085 Zinc-containing alcohol dehydrogenase superfamily, IPR002328 Zinc-containing alcohol dehydrogenase, IPR002085 Zinc-containing alcohol dehydrogenase superfamily, IPR002328 Zinc-containing alcohol dehydrogenase, IPR002085 Zinc-containing alcohol dehydrogenase superfamily, IPR002328 Zinc-containing alcohol dehydrogenase, IPR002085 Zinc-containing alcohol dehydrogenase superfamily, IPR002328 Zinc-containing alcohol dehydrogenase, IPR002085 Zinc-containing alcohol dehydrogenase superfamily, IPR002085 Zinc-containing alcohol dehydrogenase superfamily, IPR002328 Zinc-containing alcohol dehydrogenase, IPR002085 Zinc-containing alcohol dehydrogenase superfamily, IPR002328 Zinc-containing alcohol dehydrogenase, IPR002085 Zinc-containing alcohol dehydrogenase superfamily, IPR002328 Zinc-containing alcohol dehydrogenase, IPR002085 Zinc-containing alcohol dehydrogenase superfamily, IPR002328 Zinc-containing alcohol dehydrogenase, IPR002085 Zinc-containing alcohol dehydrogenase superfamily | GO:0008152 (5) metabolism |  |  | GO:0003824 (2) catalytic activity, GO:0016787 (2) hydrolase activity |
| ENSG00000165140.1, FRUCTOSE-1,6-BISPHOSPHATASE (EC 3.1.3.11) (D-FRUCTOSE-1,6-BISPHOSPHATE 1-PHOSPHOHYDROLASE) (FBPASE). [Source:SWISSPROT;Acc:P09467] | IPR000146 Inositol phosphatase/fructose-1,6-bisphosphatase |  |  | GO:0005634 (3) nucleus | GO:0005643 (2) nuclear pore, GO:0005737 (2) cytoplasm, GO:0006607 (2) NLS-bearing substrate-nucleus import, GO:0006886 (2) intracellular protein transport |
| ENSG00000168129.1, DIHYDROFOLATE REDUCTASE (EC 1.5.1.3). [Source:SWISSPROT;Acc:P00374] | IPR001796 Dihydrofolate reductase |  | GO:0005739 (4) mitochondrion | GO:0005524 (3) ATP binding | GO:0006139 (2) nucleobase, nucleoside, nucleotide and nucleic acid metabolism, GO:0009058 (2) biosynthesis, GO:0016787 (2) hydrolase activity |
| ENSG00000169910.3, ARGININOSUCCINATE LYASE (EC 4.3.2.1) (ARGINOSUCCINASE) (ASAL). [Source:SWISSPROT;Acc:P04424] | IPR000362 Fumarate lyase, IPR003031 Delta crystallin |  |  |  | GO:0000050 (2) urea cycle, GO:0006526 (2) arginine biosynthesis, GO:0016740 (2) transferase activity |
| ENSG00000171503.1, ELECTRON TRANSFER FLAVOPROTEIN-UBIQUINONE OXIDOREDUCTASE, MITOCHONDRIAL PRECURSOR (EC 1.5.5.1) (ETF-QO) (ETF-UBIQUINONE OXIDOREDUCTASE) (ETF DEHYDROGENASE) (ELECTRON-TRANSFERRING- FLAVOPROTEIN DEHYDROGENASE). [Source:SWISSPROT;Acc:Q16134] | IPR000103 Pyridine nucleotide-disulphide oxidoreductase, class-II, IPR007859 Electron transfer flavoprotein-ubiquinone oxidoreductase |  |  |  | GO:0005759 (2) mitochondrial matrix, GO:0006118 (2) electron transport, GO:0008246 (2) electron transfer flavoprotein |
| ENSG00000173599.3, PYRUVATE CARBOXYLASE, MITOCHONDRIAL PRECURSOR (EC 6.4.1.1) (PYRUVIC CARBOXYLASE) (PCB). [Source:SWISSPROT;Acc:P11498] | IPR001882 Biotin-requiring enzyme, attachment site, IPR005481 Carbamoyl-phosphate synthetase large chain, N-terminal, IPR000089 Biotin/lipoyl attachment, IPR000891 HMG-CoA lyase-like, IPR003379 Conserved carboxylase region, IPR005482 Biotin carboxylase, C-terminal, IPR005479 Carbamoyl-phosphate synthase L chain, ATP-binding, IPR001882 Biotin-requiring enzyme, attachment site, IPR005481 Carbamoyl-phosphate synthetase large chain, N-terminal, IPR000089 Biotin/lipoyl attachment, IPR000891 HMG-CoA lyase-like, IPR003379 Conserved carboxylase region, IPR005482 Biotin carboxylase, C-terminal, IPR005479 Carbamoyl-phosphate synthase L chain, ATP-binding, IPR001882 Biotin-requiring enzyme, attachment site, IPR005481 Carbamoyl-phosphate synthetase large chain, N-terminal, IPR000089 Biotin/lipoyl attachment, IPR000891 HMG-CoA lyase-like, IPR003379 Conserved carboxylase region, IPR005482 Biotin carboxylase, C-terminal, IPR005479 Carbamoyl-phosphate synthase L chain, ATP-binding |  |  | GO:0016491 (3) oxidoreductase activity | GO:0005739 (2) mitochondrion, GO:0005777 (2) peroxisome, GO:0006099 (2) tricarboxylic acid cycle, GO:0008152 (2) metabolism, GO:0009374 (2) biotin binding |
| ENSG00000177000.1, METHYLENETETRAHYDROFOLATE REDUCTASE (EC 1.5.1.20). [Source:SWISSPROT;Acc:P42898] | IPR003171 Methylenetetrahydrofolate reductase |  |  |  | GO:0004299 (2) proteasome endopeptidase activity, GO:0005634 (2) nucleus, GO:0005829 (2) cytosol, GO:0005837 (2) 26S proteasome, GO:0005839 (2) proteasome core complex (sensu Eukarya), GO:0006511 (2) ubiquitin-dependent protein catabolism |
| ENSG00000178802.3, MANNOSE-6-PHOSPHATE ISOMERASE (EC 5.3.1.8) (PHOSPHOMANNOSE ISOMERASE) (PMI) (PHOSPHOHEXOMUTASE). [Source:SWISSPROT;Acc:P34949] | IPR001230 Prenyl group binding site (CAAX box), IPR001250 Mannose-6-phosphate isomerase, type I, IPR001230 Prenyl group binding site (CAAX box), IPR001250 Mannose-6-phosphate isomerase, type I |  |  |  | GO:0005509 (2) calcium ion binding, GO:0005737 (2) cytoplasm, GO:0005975 (2) carbohydrate metabolism, GO:0016021 (2) integral to membrane |
